# Selective amino acid formulation enhances anion secretion and restores function in cystic fibrosis mutations

**DOI:** 10.3389/fphar.2025.1522130

**Published:** 2025-08-04

**Authors:** Astrid Grosche, Anusree Sasidharan, Matthias Salathe, Nathalie Baumlin, Damiano Angoli, Sreekala Prabhakaran, Xiaodong Xu, Sadasivan Vidyasagar

**Affiliations:** ^1^Department of Radiation Oncology, University of Florida, Gainesville, FL, United States; ^2^Department of Internal Medicine, Division of Pulmonary, Critical Care and Sleep Medicine, University of Kansas Medical Center, Kansas City, KS, United States; ^3^Pediatric Pulmonary Division, University of Florida, Gainesville, FL, United States

**Keywords:** cystic fibrosis, amino acids, human bronchial epithelial cells, chloride secretion, SLC26A9, CFTR

## Abstract

**Introduction:**

In cystic fibrosis (CF), most CFTR mutations cause partial (Class II) or complete (Class I) loss of function. Modulators (VX) can improve CFTR function in Class II mutations but are ineffective for Class I mutations and may cause side effects, resulting in tolerability issues with concerns about long-term safety. Apical anion secretion, essential for maintaining airway surface liquid (ASL) homeostasis, is regulated by CFTR. Alternative anion channels, like ANO1 and SLC26A9, also contribute to ASL homeostasis. Our recent work indicates that specific amino acids can modulate ion channel expression, activity, and trafficking in epithelial cells. We developed a select amino acid formulation (SAA) to enhance anion secretion in primary human bronchial epithelial cells (HBEC) with CF, regardless of mutation.

**Methods:**

Transepithelial short-circuit current was measured in wildtype (WT)- and CF-HBEC with various Class I and Class II mutations. Cells were pretreated with DMSO or VX for 24 h before apical exposure to SAA in Ussing chambers. Benzamil-insensitive current was sequentially inhibited to determine the contributions of SLC26A9, CFTR, ANO1, and NKCC1. ^36^Cl unidirectional and net fluxes (*J*_net_Cl) validated chloride secretion. Whole-cell patch-clamp studies determined the current density with SAA in WT- and CF-HBEC. CFTR, SLC26A9, and ANO1 mRNA and protein expression levels were assessed via qPCR and immunofluorescence. ASL volume, ciliary beat frequency (CBF), and mucociliary transport were also assessed.

**Results:**

SAA increased benzamil-insensitive current to 70%–85% of WT cells, and enhanced *J*_net_Cl in both Class I and II mutations. *J*_net_Cl contributed to 72%, 50%, and 39.5% of S9A13-inhibitable current in WT-, F508del^+/+^-, and G542X/R785X-HBEC, respectively. VX treatment increased current in Class II but did not affect Class I mutations. Increased chloride secretion with SAA was attributed to enhanced activity of SLC26A9 and partial CFTR restoration through elevated mRNA and membrane protein expression. SAA also increased ASL volume and CBF, confirming its effectiveness in Class I mutations.

**Discussion:**

SAA enhances chloride secretion through SLC26A9 and partial CFTR rescue in Class I and II mutations. These findings suggest SAA functions as a mutation-agnostic therapy to improve anion secretion and clinical symptoms, particularly in Class I mutations.

## Introduction

Cystic fibrosis (CF) is a rare genetic disease caused by mutations or errors in the Cystic Fibrosis Transmembrane Conductance Regulator (CFTR) gene. These mutations lead to either the absence or malfunctioning of the CFTR protein, which is essential for regulating chloride (Cl^−^) and water movement across cell membranes. This defect primarily impacts the lungs and digestive system, leading to significant health challenges. The mutation also affects the pancreas, liver, and other organs, affecting around 120,000 individuals globally. Lung impairment is the most significant consequence, as the CFTR protein is crucial for maintaining normal respiratory function. In airways, luminal fluid and electrolyte transport mechanisms maintain a thin layer of liquid, known as airway surface liquid (ASL), which covers the epithelial surface and facilitates both mucociliary clearance and local innate immunity. Volume and composition of ASL are mainly regulated by a complex interplay of sodium (Na^+^) absorption mediated by the epithelial sodium channel (ENaC) and Cl^−^ secretion facilitated by CFTR creating an osmotic gradient for net passive water movement via paracellular routes and aquaporins (Wang et al., 2014; Cutting, 2015). Additional anion and cation channels and transporters such as calcium-activated Cl^−^ channels (CaCC; syn. ANO1, TMEM16A), the solute carrier transporter SLC26A9, or ligand- and voltage-gated potassium (K^+^) channels (KCNQ, BK) are part of a well-coordinated ASL volume and composition.

The CFTR protein, part of the ATP-binding cassette transporter family, forms a small-conductance Cl^−^ channel gated by protein kinase A-mediated phosphorylation and ATP binding and hydrolysis (Pranke and Sermet-Gaudelus, 2014; Pranke et al., 2017). Only 20%–40% of nascent AA chains achieve this folded state; the rest are degraded by the endoplasmic reticulum, lysosomes, or autophagy. The CFTR protein comprises two membrane-spanning domains (MSD1 and MSD2), two nucleotide-binding domains (NBD1 and NBD2), and a central regulatory (R) domain. MSD1 and MSD2 each consist of six transmembrane segments/domains (TMD) linked by extracellular and cytoplasmic loops (CL), forming the transmembrane channel. The NBDs bind and hydrolyze ATP, while the R domain, containing serine residues phosphorylated by PKA or PKC, regulates channel gating with the NBDs (Hunt et al., 2013). The interactions between the NBDs and CLs of MSDs are critical for the proper assembly and Cl^−^ channel function (Anderson et al., 1991; Gadsby et al., 2006; He et al., 2008; Riordan, 2008; Infield et al., 2021).

Over the years, more than 2,000 gene variants have been identified, and 352 variants are associated with disease causation affecting the production, processing, and function of CFTR protein to different degrees (Mall et al., 2020; Venturini et al., 2021), with a large number of mutations impairing the posttranslational processing machinery of CFTR (Pranke and Sermet-Gaudelus, 2014). The mutations were grouped into distinct classes based on different molecular mechanisms and functional losses. This understanding provides the scientific basis for developing targeted treatments for CF. The most recent classification system groups mutations by the problems they cause in CFTR protein production. 1) Protein production mutations (Class I): A nonsense mutation resulting from premature termination codons (PTCs) or stop codons, causing premature cessation of translation with protein truncation and loss of function; 2) Protein processing mutations and folding in the Golgi (Class II): Includes the common F508 deletion mutation, where CFTR is retained in the endoplasmic reticulum and eventually degraded; 3) Gating mutations (Class III): CFTR is resistant to phosphorylation or ATP binding, preventing the CFTR channels from staying open and conducting Cl^−^/bicarbonate (HCO_3_^−^); 4) Conduction mutations (Class IV): The mutation alters the inside of the channel so that Cl^−^/HCO_3_^−^ movement is reduced despite phosphorylation of CFTR; 5) Insufficient protein mutations (Class V): Arise from splicing machinery defects, generating both aberrantly and correctly spliced transcripts with reduced amount of normal CFTR protein at the cell surface (Boyle and De Boeck, 2013; Wang et al., 2014). Among the different classes of CFTR mutations, Class I, II and III cause a lack of or minimal residual CFTR function resulting in a severe CF phenotype. Most mutant nascent polypeptide chains do not pass quality control in the endoplasmic reticulum, but the protein that traffics to the plasma membrane remains unstable, only partially functional, and is rapidly endocytosed and degraded by the proteasomal pathway or autophagy (Serohijos et al., 2008). In nonsense mutation stop codons (UGA, UAG, UAA) terminate translation, while premature termination codons (PTCs) (e.g., TGA, TAG, TAA) occur in normal coding sequence due to single base pair substitutions and are typically destroyed by nonsense-mediated mRNA decay, though some truncated proteins may still form (Chang et al., 2007). Translation can continue past stop codons via mechanisms like ribosomal frameshifting, suppressor tRNAs, or natural read-through (Beier and Grimm, 2001; Ko et al., 2022). Suppressor tRNAs can outcompete termination factors, enabling amino acid incorporation (Beznosková et al., 2021). Pharmacological agents (e.g., aminoglycosides, dipeptides, oxadiazoles) can enhance PTC read-through, restoring full-length protein production despite nonsense mutations (Dabrowski et al., 2018). The functional outcome depends on PTC location: C-terminal PTCs (e.g., R1162X in NBD2) often yield poorly functional CFTR, while N-terminal PTCs (e.g., G542X in NBD1) may produce structurally altered proteins (Yeh and Hwang, 2020).

Since the discovery of the CFTR gene in 1989, significant progress has been made in developing small-molecule CFTR mediators affecting CFTR function (Ghelani and Schneider-Futschik, 2020). CFTR modulators, such as the correctors that improve CFTR protein folding and potentiators increasing CFTR function, have transformed treatment for many CF mutations. These treatments are effective for Class II-V mutations but do not address Class I mutations. A triple-combination of CF modulators (VX770, VX445, and VX661; VX) that target the NBD1 - TMD1 interface and NBD2 of the misfolded protein and restores approximately 62% of F508del channel function (Veit et al., 2020). Approved by the FDA in 2019, VX was the first drug for treating CF individuals with homozygous for F508del or compound F508del heterozygous with minimal function, showing clinically meaningful improvements in lung function and quality of life (Cuevas-Ocaña et al., 2020). Approximately 10%–15% of CF patients who carry nonsense or PTC mutations do not benefit from VX (Fajac and Sermet, 2021). Additionally, off-target pharmacology often results in tolerability issues, raising concerns about long-term safety, especially in children under 12 years (Heneghan et al., 2023). Thus, there is a need to develop therapies that are safe, effective, and work for Class I mutations, or are mutation-agnostic.

Among the five classes of CFTR mutations, Class I, II and III cause a lack of or minimal residual CFTR function resulting in a severe CF phenotype. Deletion of the AA phenylalanine at position 508 (F508del) from NBD1 of the CFTR protein disrupts both protein folding and function. Most mutant nascent polypeptide chains do not pass quality control in the endoplasmic reticulum, but the protein that traffics to the plasma membrane remains unstable, only partially functional, and is rapidly endocytosed and degraded by the proteasomal pathway or autophagy (Serohijos et al., 2008).

Since the discovery of the CFTR gene in 1989, significant progress has been made in developing small-molecule CFTR mediators affecting CFTR function (Ghelani and Schneider-Futschik, 2020). A triple-combination of CF modulators (VX770, VX445, and VX661; VX) that target the NBD1 - TMD1 interface and NBD2 of the misfolded protein and restores approximately 62% of F508del channel function (Veit et al., 2020). Approved by the FDA in 2019, VX was the first drug for treating CF individuals with homozygous for F508del or compound F508del heterozygous with minimal function, showing clinically meaningful improvements in lung function and quality of life (Cuevas-Ocaña et al., 2020). In this study, VX and forskolin treatment rescued 69.8% of the CFTR function in F508del^+/+^-HBEC compared to WT-HBEC, aligning with previous findings (Veit et al., 2020). However, CF-HBEC with heterozygous or homozygous nonsense mutations showed less or no improvement in CFTR function.

Approximately 10%–15% of CF patients who carry nonsense or PTC mutations do not benefit from VX (Fajac and Sermet, 2021). Nonsense mutations, caused by PTCs or stop codons, lead to protein truncation and loss of function, classifying them as Class I or protein production mutations. Stop codons (e.g., UGA, UAG, UAA) signal the end of protein-coding sequences in mRNA. Conversely, PTCs (e.g., TGA, TAG, TAA) occur in normal coding sequences due to single base pair substitutions and are typically destroyed by nonsense-mediated mRNA decay, although some truncated polypeptides are produced from residual mRNA (Chang et al., 2007). Termination suppression mechanisms include ribosomal frameshifting, suppressor tRNAs, and natural stop codon read-through, which can promote further translation (Dabrowski et al., 2015). Certain tRNAs can infrequently result in accommodation of near-cognate tRNA resulting in translational read-through of the stop codon. Suppressor tRNAs, which outcompete translation termination factors, drive the incorporation of cognate amino acids (Beier and Grimm, 2001; Ko et al., 2022). Thus, tRNAs control every translational phase (Beznosková et al., 2021). Pharmacological compounds such as aminoglycosides, dipeptides, or oxadiazoles can stimulate PTC read-through or suppress nonsense mutations, facilitating cognate AA-tRNA incorporations (Dabrowski et al., 2018). This can result in the production of full-length proteins even in the presence of nonsense mutations. However, unintended amino acid insertions during read-through can lead to missense mutations at the PTC site (Xue et al., 2017). The search for read-through agents is complicated by the position of PTCs affecting CFTR function. PTCs near the C-terminus, such as R1162X with PTC at NBD2, result in poorly functional truncated or read-through full-length CFTR proteins. In contrast, early sequence PTCs, like G542X with PTC at NBD1, yield read-through products with missense mutations and structural modifications (Yeh and Hwang, 2020).

Amino acids regulate cell functions beyond being nutrients and protein building blocks. They influence gene expression, mRNA levels, tRNA function, cell signaling, metabolism, antioxidant responses, and immune functions (Wu, 2013). Charging tRNAs ensures the correct AA attaches to its corresponding tRNA by aminoacyl-tRNA synthetases, a process influenced by AA availability, which affects translation accuracy and efficiency. Modifications in tRNAs, affected by AAs, impact codon recognition fidelity, including near-cognate and non-cognate interactions. SAA-induced anion *I*_sc_ (benzamil-insensitive *I*_sc_) was observed in F508del^+/+^-HBEC and HBEC with CFTR protein production mutations (Class I).

Recent studies from our laboratory using specific amino acids (AAs) have shown to regulate anion channels, transporters, intestinal motility, and tight junctions (Yin et al., 2014; Yin et al., 2016; Gupta et al., 2020). Amino acids in addition to their well-known role in metabolism and nutrition have recently provided increasing evidence of their effect on a wide variety of key regulatory and signaling effects on cellular homeostasis, proliferation, survival, antioxidative defenses, and immune responses (Kimball and Jefferson, 2004; Brasse-Lagnel et al., 2010). AAs influence gene expression, mRNA levels, tRNA function, cell signaling, metabolism, antioxidant responses, and immune functions (Wu, 2013; Broer and Broer, 2017). Additionally, a process influenced by AA availability can affect translation accuracy and efficiency. In this study, the AAs were used to identify and regulate key functions altered in CF by selectively targeting disease-specific signaling pathways and molecular mechanisms. The study aimed to identify specific AAs that stimulate anion channel-mediated anion secretion in primary human bronchial epithelial cell (HBEC) cultures derived from donor lungs with various CF mutations, leading to increased fluid secretion. This was measured by increased ASL, enhanced ciliary beat frequency (CBF), and mucociliary clearance.

## Materials and Methods

### Human bronchial epithelial cell cultures and treatments

Normal primary HBEC (Wildtype; WT) from two donors (passage 1) were obtained from the University of Alabama CF Research and Translation Core Center under a Material Transfer Agreement with the University of Florida (UAB A-302178; IRB 00000726). Primary HBEC with the homozygous Class II mutation F508del^+/+^ were harvested from a donor lung (Pediatric tissue and data bank; IRB #201602392) under regulations of the Pediatric and Adult Pulmonary Center at the University of Florida. Primary HBEC (passage 2) with the heterozygous Class I mutations G542X/R785X and W1282X/R1162X were received from Dr. S. Randell, Marsico Lung Institute, University of North Carolina, Chapel Hill, NC, United States, and primary HBEC (passage 2) with the homozygous Class I mutation G542X^+/+^ were a gift from Dr. B. Bridges, Rosa Franklin University, Chicago, IL, United States. Because the cells were obtained from deceased individuals with minor, de-identified information, their use does not constitute human subjects research as defined by CFR 46.102. The providers assured that the cells were acquired in accordance with the Common Rule, and the projects were approved by the Institutional Review Boards of the University of Alabama, the University of North Carolina, and Rosa Franklin University. A written consent was obtained before procurement of the cells. All experiments were performed by the guidelines and regulations described by the Declaration of Helsinki and the Huriet-Serusclat and Jardet law on human research ethics, and the protocols to obtain, culture, store, and study HBEC were approved by the Institutional Review Board of the University of Florida (IRB #201602392).

Primary HBEC were expanded in expansion media (PneumaCult Ex Plus; StemCell Technologies, United States), and cells of passage 3 were seeded on collagen IV-coated (Sigma, United States) permeable snapwell or transwell inserts (12 or 24 mm, 0.4 µM pore polyester membrane; Corning Costar, United States) at 5 × 10^5^ cells·cm^−2^ in expansion medium containing 1% penicillin/streptomycin and kept at 37°C and 5% CO_2_. After cells reached 95% confluence, cells were differentiated in PneumaCult ALI medium (StemCell Technologies, United States) containing 1% penicillin/streptomycin and 2% Ultroser G (Crescent Chemical Co., United States) at an air-liquid interface. The ALI medium was changed every 2 days until cells were fully differentiated (25–30 days). Differentiated HBEC, characterized by cilia motility, were either treated with DMSO (max. of 0.05%) or with a combination of the CFTR correctors VX661 (tezacaftor, 18 μM; MedChemExpress, United States, cat # HY-15448), and VX445 (elexacaftor, 3 μM; cat # HY-111772), and the potentiator VX770 (ivacaftor, 1 μM; cat # HY-13017) for 24 h prior to the experiments (Keating et al., 2018).

### Ussing chamber experiments and flux studies

Differentiated WT- and CF-HBEC growing on snapwell inserts were mounted in pre-warmed, calibrated Ussing chambers (VCC MC8; Physiologic Instruments, United States). Each side was bathed in 5 mL Ringer’s solution containing (mM): 113.8 Na^+^, 93.6 Cl^−^, 25 HCO_3_^−^, 5.2 K^+^, 2.4 HPO_4_^−^, 0.4 H_2_PO_4_^−^, 1.2 Mg^2+^, 1.2 Ca^2+^, and 75 mannitol, with an osmolarity of 300 mOsm and a pH of 7.4. The basolateral bathing solution contained 5 mM of glucose. Chambers were bubbled with 95% O_2_ and 5% CO_2_ and maintained at 37°C. After a 30-min equilibration period, transepithelial short circuit current (*I*_sc_, in µeq·h^−1^·cm^−2^) and transepithelial electrical resistance (TEER) were recorded every 30 s while continuously clamping the membrane potential to zero. Voltage clamping eliminated passive ion movements across the membranes due to electrochemical potential gradients, osmotic gradients, and hydrostatic forces. Any current recorded under zero membrane potential was due to active ion movements, represented as *I*_sc_.

To measure the anion current, apical ENaC activity was blocked using 6 µM benzamil (Tocris, United States) for 15 min, and the benzamil-insensitive *I*_sc_ was recorded. In one set of experiments, CFTR was stimulated using 10 µM forskolin (Cayman Chemical, United States) added to the apical and basolateral sides of the Ussing chambers. This was followed by sequential inhibition of CFTR using 20 µM CFTRinh-172 (Tocris, United States), and anoctamin (ANO1) inhibition using 10 µM CaCCinh-A01 (Tocris, United States) added to the apical and basolateral sides of the Ussing chambers. Any remaining anion current was inhibited using 20 µM bumetanide (Tocris, United States), a Na–K–2Cl (NKCC1) cotransporter inhibitor added to the basolateral sides. Bumetanide prevents basolateral uptake of Cl^−,^ which is essential for its apical exit via anion channels. In another set of experiments, the contribution of SLC26A9 to the benzamil-insensitive anion *I*_sc_ was evaluated by adding the specific SLC26A9 inhibitor, S9A13 (10 µM), to the apical side of the chamber before sequential inhibition of CFTR, ANO1, and NKCC1. The SLC26A9 inhibitor was kindly provided by Dr. Wan Namkung and Dr. Ikyon Kim from the College of Pharmacy, Yonsei Institute of Pharmaceutical Sciences, Yonsei University, Incheon, South Korea (Jo et al., 2022). An additional set of cells that did not receive forskolin or S9A13 during the initial 15-min recording of the benzamil-insensitive *I*_sc_ served as controls for the response patterns. Changes in *I*_sc_ responses (delta *I*_sc_) were calculated by subtracting the *I*_sc_ recordings taken after 15 min of inhibitor or stimulator addition from those taken before their addition.

In the initial experiments, CF-HBEC (F508del^+/+^ and G542X^+/+^) were exposed to single AAs added to the apical and basolateral side of the Ussing chambers at a concentration of 8 mM. AAs were ranked based on their effect on benzamil-sensitive, benzamil-insensitive, forskolin-stimulated, CFTRinh-172-, CaCCinh-A01-, and bumetanide-sensitive *I*_sc_. The selected AAs that showed the highest increase in benzamil-insensitive anion *I*_sc_ and a decrease or unchanged benzamil-sensitive *I*_sc_ were L-glycine, L-cysteine, L-proline, L-tyrosine, and L-lysine. These AAs were further evaluated for their transport characteristics. The response of the selected AAs on benzamil-insensitive *I*_sc_ was maximized by optimizing the AA concentrations using saturation kinetic studies in CF-HBEC. Briefly, HBEC were bathed in Ringer’s solution, ENaC was blocked with benzamil, and increasing concentrations of AAs were added to the apical side at 3-min intervals, until benzamil-insensitive *I*_sc_ saturated. Mannitol was added to the basolateral side at the same concentration to compensate for any osmotic gradients. The maximum effect of AAs on benzamil-insensitive *I*_sc_ (*V*_max_), the Hill constant (*K*_M_), and the Hill coefficient *n* were used to evaluate individual AA performance, AA transport characteristics, and optimal AA concentrations for maximum benzamil-insensitive *I*_sc_.

After optimizing the concentrations, the selected AAs (SAA) were formulated as follows (mM): 30 L-glycine, 22.5 L-cysteine, 15 L-proline, 1.2 L-tyrosine, and 1 L-lysine (Ajinomoto, United States), diluted in Ringer’s solution containing (mM): 113.8 Na^+^, 93.6 Cl^−^, 25 HCO_3_^−^, 5.2 K^+^, 2.4 HPO_4_^−^, 0.4 H_2_PO_4_^−^, 1.2 Mg^2+^, 1.2 Ca^2+^ at pH 7.4, and adjusted to 300 mOsm with mannitol. This optimized SAA formulation was subsequently utilized in the experiments described in this study.

Chloride secretion was evaluated using unidirectional and net ^36^Cl isotope flux experiments in Ussing chambers with the membrane potential continuously clamped to zero. Transepithelial net Cl^−^ flux (*J*_net_Cl) was calculated for WT-HBEC and CF-HBEC with various mutations. Unidirectional fluxes for Cl^−^ from the apical-to-basolateral (*J*_ms_) and the basolateral-to-apical sides (*J*_sm_) were measured, and the net ion flux across the cell culture was calculated using the formula *J*_ms_Cl - *J*_sm_Cl = *J*_net_Cl. The results were expressed as µeq·h^−1^·cm^−2^. After blocking ENaC activity with 6 µM benzamil, HBEC were paired based on a similar TEER. Chloride isotopes (^36^Cl) were added to either the basolateral or apical side (hot side) of each pair, and samples were taken every 15 min from the contralateral sides (cold side) and replaced by fresh bath solution. ^36^Cl activities were analyzed in a liquid scintillation counter (LS6500 Multipurpose Scintillation Counter, Beckman Coulter).

### Patch clamp recordings

Differentiated WT- and CF-HBEC (F508del^+/+^, and G542X/F785R) were seeded on glass coverslips in ALI medium, maintained for 24–48 h, and then treated with DMSO or VX for 24 h before electrophysiological recordings. Coverslips were placed on the stage of an inverted microscope with a bath perfusion chamber. The internal pipette solution consisted of (mM): 20 KCl, 120K-Gluconate, 5 HEPES, 0.2 EGTA, 0.5 MgCl_2_. The bath Ringer’s solution consisted of (in mM): 113.8 Na^+^, 93.6 Cl^−^, 25 HCO_3_^−^, 5.2 K^+^, 4.8 HPO_4_^−^, 0.4 H_2_PO_4_, 1.2 Mg^2+^, 1.2 Ca^2+^ and 75 mannitol, which was bubbled with 95% O_2_ and 5% CO_2_. The electrolyte mixture for the AA formulation (SAA) consisted of (mM): 30 L-glycine, 22.5 L-cysteine, 15 L-proline, 1.2 L-tyrosine, and 1 L-lysine, diluted in Ringer’s solution containing (mM) 113.8 Na^+^, 93.6 Cl^−^, 25 HCO_3_^−^, 5.2 K^+^, 2.4 HPO_4_^−^, 0.4 H_2_PO_4_^−^, 1.2 Mg^2+^, 1.2 Ca^2+^ at pH 7.4, and adjusted to 300 mOsm with mannitol. The current density was calculated as the amount of current (in picoamp; pA) carried through the cell membrane after normalizing to the cell size (in picofarad; pF) and expressed as pA/pF. To evaluate the amount of CFTR current blocked by CFTRinh-172 (10 µM), the current density was set at minus 80 mV, and sets of recordings were performed using a voltage clamp ramp protocol from minus 80 mV to plus 80 mV with a holding potential of minus 10 mV.

### Real-time quantitative PCR analysis

Differentiated WT- and CF-HBEC grown on 12 mm transwell filters were treated with DMSO or VX for 24 h, followed by apical exposure to Ringer’s solution or SAA for 4 h at 37°C and 5% CO_2_. After incubation, the cells were washed with ice-cold PBS and harvested from the inserts using a cell scraper. The cell suspension was incubated in 1 mM dithiothreitol diluted in PBS for 20 min, then washed three times with ice-cold PBS, with each wash followed by centrifugation at 1,200 × *g* for 5 min. After the final centrifugation, lysis buffer (RNeasy Mini Kit; Qiagen, United States) was added to the cell pellet and vortexed for 2 min before further processing according to manufacturer’s instructions. The mRNA concentration was determined by using the spectrophotometric nanodrop method (Agilent BioTek Epoch, United States), and cDNA synthesis was performed using the iScript cDNA Synthesis Kit (Bio-Rad, United States) following manufacturer’s instructions. A total of 1,000 ng of mRNA was reverse transcribed to cDNA using a cDNA Synthesis kit (BioRad, United States). Quantitative PCR reactions were performed in duplicates from four independent experiments using 10 ng of mRNA-equivalent cDNA, the CFX-Connect Real-Time PCR detection system (Bio-Rad, United States), SsoAdvanced Universal SYBR Green Supermix (Bio-Rad, United States), and primers (250 nM) at their optimum melting and annealing temperatures. The following primers were used: SLC26A9 (FW: CGT​GGT​AGA​CAG​AGC​CGC​AT and Rev: GGC​TGA​GGA​ACA​TCT​GAA​GGC), ANO1 (FW: GAG​CCA​AAG​ACA​TCG​GAA​TCT​G, and Rev: TGA​AGG​AGA​TCA​CGA​AGG​CAT), CFTR (FW: GCG​AAG​ATC​TTG​CTG​CTT​GA, and Rev: CTG​CCG​CAC​TTT​GTT​CTC​TT), and RPS13 (FW: CGA​AAG​CAT​CTT​GAG​AGG​AAC​A, and REV: TCG​AGC​CAA​ACG​GTG​AAT). To identify the read-through mRNA, a CFTR primer at the 3′end of all the mutations was used. The cycle numbers at the linear range of the amplification curve were used for the calculation. Relative quantification of mRNA expression was determined using the Pfaffl method (Pfaffl, 2001), with RPS13 as the reference gene. The mRNA levels were expressed as fold change relative to WT-HBEC for all genes studied. Melt peaks were analyzed to confirm specific primer binding.

### Immunofluorescence

Differentiated WT- and CF-HBEC grown on 12 mm transwell filters were treated with DMSO or VX for 24 h, followed by apical exposure to 200 µL of Ringer’s solution or SAA for 1 hour at 37°C and 5% CO_2_. Cells were then fixed in 4% paraformaldehyde and embedded in paraffin. Cross-sections (4 µm) were mounted on silane-coated glass slides, deparaffinized, rehydrated, and heat pre-treated in retrieval buffer at pH 6.0 (Biocare Medical, United States) as previously described (Sasidharan et al., 2024). After blocking with Dako Protein Block, sections were incubated overnight at 4°C with the following antibodies: mouse monoclonal anti-CFTR (clone 596; Dr. Martina Gentzsch, CFTR Antibody Distribution Program, cat # 596; RRID: AB_2923486), rabbit polyclonal anti-TMEM16 A (Abcam, cat # ab53212; RRID: AB_883075) and rabbit polyclonal anti-SLC26A9 (LSBio, cat # LS-C682325; RRID: AB_2923487) diluted in Dako Antibody Diluent (1:100). Secondary antibodies, goat anti-mouse superclonal recombinant antibody conjugated with AlexaFluor488 (ThermoFisher, cat # A28175; RRID: AB_2536161) or goat anti-rabbit superclonal recombinant antibody conjugated with AlexaFluor647 (ThermoFisher, cat # A27040; RRID: AB_2536101) were used at a concentration of 1 μg/mL incubated for 1 hour at room temperature. Nuclei were stained with DAPI for 15 min, and cells were mounted in an aqueous mounting medium before analysis. Signals were analyzed at ×600 magnification using the Laser Scanning Olympus Fluoview FV1000 confocal microscope.

### Airway surface liquid

To simulate inhalation therapy as a treatment option for SAA-mediated epithelial cell secretion, 200 µL of Ringer’s solution or SAA was nebulized onto the surface of WT-HBEC and Class I-HBEC (G542X/R785X) culture inserts using the Vitrocell Cloud exposure system (Vitrocell, Germany), as described by Chung et al. (2019). Based on the treatment volume, the nebulizer surface area (approx. 116.5 cm^2^), the filter surface area (1.12 cm^2^), and the AA concentrations in SAA (70 mM), approximately 20–30 mM of AAs were deposited onto each culture filter. Immediately after nebulization (0 h) and 4 h later, high-resolution images of the cell cultures were used to estimate ASL volumes using meniscus scanning (Harvey et al., 2011). Briefly, the HBEC culture plate was placed on an Epson scanner without the lid, at room temperature, and with a humidity of at least 50%–60% to prevent dehydration during recording. Data were analyzed using ImageJ and Dr. Myerburg’s software. ASL volume was estimated based on the meniscus light refraction, and delta ASL at 4 h was calculated, representing the difference between the 0-h and 4-h measurements, with a positive value indicating ASL secretion.

### Ciliary beat frequency

Ciliary beat frequency of nebulized WT- and Class I-HBEC (G542X/R785X) was recorded at 0 h (immediately after nebulization) and 4 h post-nebulization using a high-speed Basler acA645 camera (Basler, Ahrensburg, Germany) mounted on a Zeiss Axiovert 200 M (Carl Zeiss, Jena, Germany) running SAVA software (Sisson et al., 2003), as previously described (Salathe and Bookman, 1999). Briefly, the HBEC culture plate was mounted on the microscope stage at room temperature, and five videos were recorded for each culture at designated time points.

### Mucociliary transport (MCT)

WT- and Class I-HBEC (G542X/R785X) grown on 12 mm transwell inserts were nebulized with 200 μL Ringer’s solution or SAA 4 h before recording MCT (Sears et al., 2015). Briefly, 10 µL of 1-µm fluorescently labeled carboxylate-modified polystyrene microbeads (Fluospheres; ThermoFisher, United States) were added to the culture surface at a 1:10,000 dilution. The velocity of the beads was recorded every 3 s for 30 s at an emission of 515 nm. The MCT speed was estimated (μm/s) using the Manual Tracking ImageJ plugin.

### Statistical analysis

Results are presented as mean ± standard error of the mean (SEM), and analyses were performed using the OriginPro 2021 software package. For saturation kinetics, OriginPro software was used to calculate *K*_M_, *V*_max,_ and the Hill coefficient *n* via the Hill1 equation. The Shapiro-Wilk test assessed normal distribution for each treatment group. If normally distributed, a two-sample t-test compared the overall effect of Ringer’s solution and SAA on measured parameters, while a paired t-test compared values before and after adding inhibitors, and ASL and CBF values between two time points. For multi-sample comparisons (Ringer, SAA, and VX), one-way ANOVA with *post hoc* Bonferroni was used for pairwise comparison. For non-normally distributed samples, the Kruskal–Wallis test followed by the Mann-Whitney U test was used. A P-value <0.05 was considered statistically significant.

## Results

### Selection of amino acids that increase anion current and decrease ENaC current

**FIGURE 1:**
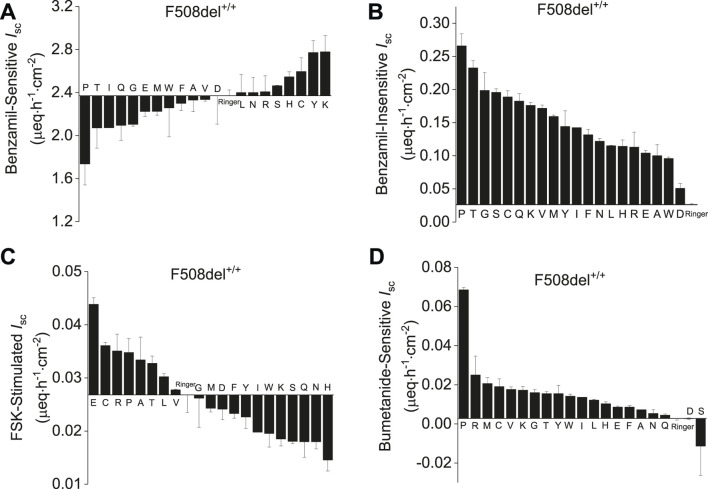
Ranked evaluation of amino acids based on their effect on benzamil-sensitive, -insensitive, forskolin-stimulated and bumetanide-sensitive *I*_sc_ in CF-HBEC with the F508del^+/+^ mutation mounted in Ussing chambers. All amino acids were used at 8 mM concentration except for tyrosine (1.2 mM) due to its limited solubility in Ringer’s solution. **(A)** Benzamil-sensitive *I*_sc_ in F508del^+/+^-HBEC exposed to individual amino acids. AAs displaying *I*_sc_ values lower than Ringer’s solution also exhibited reduced benzamil-sensitive *I*_sc_ and therefore promoting a hyperpolarized state in the HBEC. **(B)** Benzamil-insensitive *I*_sc_ recorded in F508del^+/+^-HBEC exposed to individual amino acids represents the total anion current. All the AAs studied elicited an anion current higher than that observed in cells bathed in Ringer’s solution. **(C)** Forskolin (FSK)-stimulated *I*_sc_ measurements in F508del^+/+^-HBEC exposed to individual amino acids revealed that only glutamic acid, cysteine, arginine, proline, alanine, tyrosine, leucine and valine were the only amino acids that exhibited increased *I*_sc_ in the presence of forskolin. **(D)** Bumetanide-sensitive *I*_sc_in F508del^+/+^-HBEC exposed to individual amino acids revealed that bumetanide inhibited the anion current for all the amino acids tested except for serine and aspartic acid. The values are from n = 2–6 separate experiments and presented as mean ± SEM.

Twenty AAs were screened at 8 mM concentration by adding respective AAs to the apical and basolateral side of CF-HBEC (F508del^+/+^, and G542X^+/+^) mounted in Ussing chambers and ranked based on benzamil-sensitive and -insensitive *I*_sc_ (Figures 1A,B). The absence of functional CFTR disrupts anion secretion, leading to hyperpolarization of the HBEC. This hyperpolarization enhances ENaC-mediated electrogenic cation *I*_sc_ as the cell attempts to counteract the lost anion function. Since hyperpolarization is essential for activating potential anion channels, it is critical to maintain a low ENaC-mediated cation *I*_sc_ to ensure effective anion secretion. CF-HBEC with the F508del^+/+^mutation were used to screen AAs based on their ability to increase CFTR or other anion channel-mediated *I*_sc_ and decrease benzamil-sensitive (ENaC) *I*_sc_. CFTR and/or anion channel activity was increased using forskolin. Because the basolateral Cl^−^ uptake mediated by the Na-K-2Cl cotransporter 1 (NKCC1) is crucial for apical anion secretion in HBEC, inhibiting NKCC1 with bumetanide (Figures 1C,D) indirectly measures apical anion channel activity. In addition, CF-HBEC with the Class I mutation G542X^+/+^ were used to rank individual AAs based on their effects on both benzamil-sensitive and -insensitive *I*_sc_. These cells were also employed to assess changes in *I*_sc_ resulting from sequential inhibition of CFTR, ANO1, and NKCC1 using CFTRinh-172, CaCCinh-A01, and bumetanide, respectively (Supplementary Figures S1A–E). Subsequently, the AAs proline, glycine, cysteine, lysine, and tyrosine were selected for their ability to decrease benzamil-sensitive *I*_sc_ and increase the anion *I*_sc_.

### Saturation kinetic studies on selected amino acids

**FIGURE 2:**
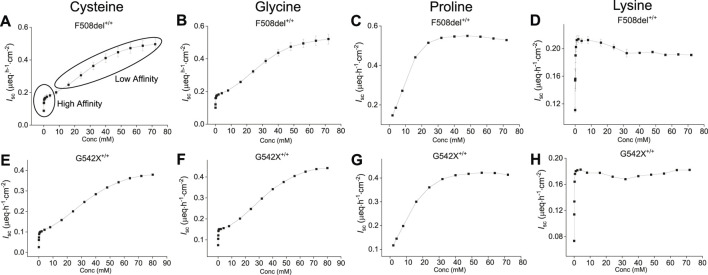
Representative saturation kinetic plots for selected amino acids—cysteine, glycine, proline, and lysine—in HBEC with two different CF mutations (top: F508del^+/+^, bottom: G542X^+/+^). Shown are dose-dependent changes in benzamil-insensitive short-circuit current (*I*_sc_) in response to increasing apical concentrations of: **(A, E)** Cysteine, **(B, F)** Glycine, **(C, G)** Proline, and **(D, H)** Lysine. Cysteine and glycine exhibited early saturation of *I*_sc_ at lower concentrations, suggesting uptake via a high-affinity transporter, followed by continued increase in *I*_sc_ at higher concentrations, consistent with an additional low-affinity transport system responsible for the anion current. The saturation kinetics of proline and lysine suggest they are each transported by a single transport system. Each plot represents data from two independent experiments per donor. Values are presented as mean ± SEM.

To evaluate the AA concentration**-**dependent response on benzamil-insensitive *I*_sc_, increasing concentrations of selected AAs (cysteine, glycine, proline, lysine and tyrosine) were added to the apical side of CF-HBECs (F508del^+/+^, and G542X^+/+^) at 3-min intervals. As a result, a dose-dependent increase in benzamil-insensitive *I*_sc_ was observed for cysteine, glycine, proline, and lysine in both CF mutations (Figure 2). Due to tyrosine’s limited solubility its response did not exhibit a dose-dependent saturable kinetic profile. Cysteine and glycine exhibited early signs of saturation at lower concentrations, yet *I*_sc_ continued to increase with higher AA concentration. This biphasic pattern suggests involvement of more than one apical AA transporter. In contrast, the AAs proline and lysine showed saturation consistent with a single transporter (Figures 2C,D,G,H). The early saturation therefore represents a high-affinity transporter with a low *K*_M_ and low Vmax, whereas continued increases at higher AA concentrations reflect a secondary low-affinity transporter with high *K*_M_ and high V_max_. The sigmoidal nature of these saturation curves is characteristic of ion-coupled transporters and allosteric enzyme kinetics. Kinetic parameters—*K*_M,_ V_max_, and the Hill coefficient n—were derived using the Hill1 equation (Lolkema and Slotboom, 2015). The hill coefficient *n* provides additional information on AA-transporter binding characteristics with *n* > 1 suggesting two or more binding sides or protein subunits, and positive cooperativity for substrate binding (binding of one substrate facilitates binding of another substrate). In contrast, *n* = 1 refers to a single substrate binding site or multiple binding sites that do not interact cooperatively, and *n* < 1 indicates negative cooperativity for substrate binding.

Based on saturation kinetics, cysteine appears to utilize at least two apical transport systems in both mutations (Figures 2A,E); Supplementary Figures S2A,B and S3A,B). In F508del^+/+^-HBEC, a high-affinity transporter with a *K*_M_ of 0.12 mM, *V*_max_ of 0.2 µeq·h^−1^·cm^−2^, and *n* of 0.45 suggested a single substrate binding side with negative cooperativity for multiple substrate bindings. The second low-affinity transporter with a *K*_M_ of 32.3 mM and *V*_max_ of 0.56 µeq·h^−1^·cm^−2^ and *n* of 2.15 indicated a positive substrate binding cooperativity or ion-coupled transport mechanism with two or more substrate binding sides (Supplementary Figures S2A,B).

Glycine transport showed similar properties (Supplementary Figures S2C,D: for F508del^+/+^; Supplementary Figures S3C,D: for G542X^+/+^) with one high-affinity transporter at *K*_M_ of 0.07 mM, *V*_max_ of 0.2 µeq·h^−1^ cm^−2^ and *n* of 0.7 in F508del^+/+^-HBEC, while the second transporter had low-affinity binding features with *K*_M_ of 30.5 mM, *V*_max_ of 0.6 µeq·h^−1^ cm^−2^ and n of 2.3 at higher glycine concentrations suggesting the involvement of more than one apical AA transporter for glycine. In contrast, the proline transporter required lower substrate concentrations than cysteine and glycine at similar *V*_max_ (F508del^+/+^: 0.6 µeq·h^−1^ cm^−2^ with *K*_M_ of 10.8 mM and *n* of 2.8 and G542X^+/+^: 0.4 µeq h^−1^ cm^−2^ with *K*_M_ of 13.3 mM and *n* of 2.1) indicating a different transport mechanism with positive substrate-binding cooperativity and potentially two or more binding sides (Figures 2C,G). The response of lysine was mediated at low concentrations through a high-affinity, positive cooperative substrate binding transporter with a *K*_M_ of 0.14 mM, *V*_max_ of 0.2 µeq·h^−1^ cm^−2^, and *n* of 1.5 in F508del^+/+^-HBEC and *K*_M_ of 0.09 mM, *V*_max_ of 0.2 µeq h^−1^ cm^−2^, and *n* of 1.4 in G542X^+/+^-HBEC. Thus, lysine contributed only marginally to the overall anion-secretory response of SAA (Figures 2D,H: for both mutations). Due to its low solubility, tyrosine saturation kinetics were not performed. The concentrations of AAs used in the formulation were selected based on obtaining high anion *I*_sc_ (*V*_max_) and solubility of the respective AA. Cysteine, proline, glycine, lysine, and tyrosine were used at 22.5, 15, 30, 1, and 1.2 mM in the formulation.

### Dose optimization using saturation kinetics of SAA

**FIGURE 3:**
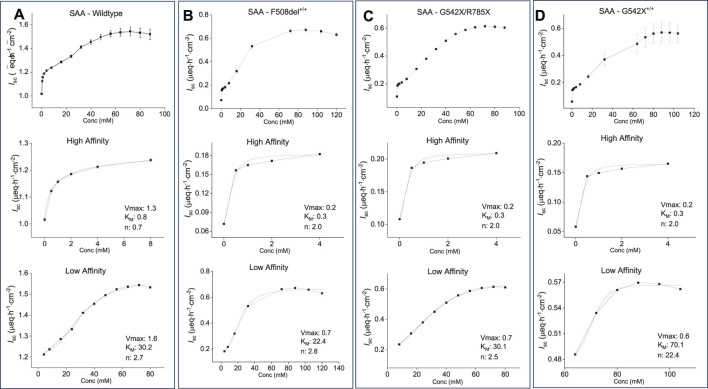
Representative saturation kinetic plots and saturation kinetic parameters for the selected amino acid formulation (SAA) in wildtype (WT) and CF-HBEC with different mutations. The Hill1 equation was used to calculate the maximum effect (V_max_), substrate-transporter affinity (*K*_M_), and cooperativity of substrate binding (Hill coefficient, n) based on changes in benzamil-insensitive short-circuit current (*I*_sc_). Saturation curves show consistently a sigmoidal pattern across all donors and mutations (top), with early *I*_sc_ saturation at lower concentrations indicating high-affinity transport (middle), followed by continued *I*_sc_ increases at higher concentrations, consistent with low-affinity transport (bottom). Each transport phase was defined by distinct V_max_, *K*_M_, and Hill coefficient values. **(A)** WT-HBEC, **(B)** F508del^+/+^, **(C)** G542X/R785X, and **(D)** G542X^+/+^. Each donor was tested in two independent experiments. Values are presented as mean ± SEM.

To evaluate dose-response and determine the *K*_M_ and *V*_max_ of the formulation, increasing concentrations of the combination of all five AAs (SAA) were applied to the apical side of WT- and CF-HBEC with various mutations. The response followed a sigmoidal saturation curve in WT- (Figure 3A) and CF-HBEC with F508del^+/+^ (Figure 3B), G542X/R785X (Figure 3C), and G542X^+/+^ (Figure 3D), similar to that observed with cysteine and glycine. At low SAA concentrations, an initial saturation point was observed, consistent with a high-affinity transport mechanism seen with cysteine, glycine, and lysine. With further increases in SAA concentration, a second peak in *I*_sc_ was reached, with a *V*_max_ of 1.6 µeq h^−1^ cm^−2^ in WT-HBEC and 0.6–0.7 µeq h^−1^ cm^−2^ in CF-HBEC. The final formulation concentration was selected based on the AA concentrations that produced maximal anion secretion (*V*_max_). Notably, the formulation altered *K*_M_ in different CF genotypes: *K*_M_ decreased in F508del^+/+^-HBEC, increased in G542X^+/+^-HBEC, and remained unchanged in G542X/R785X-HBEC compared to WT-HBEC. These findings were used to optimize the formulation concentration for maximal anion secretion.

### SAA induced anion secretion while Na^+^ absorption remained unchanged or decreased across all CF mutation classes

**FIGURE 4:**
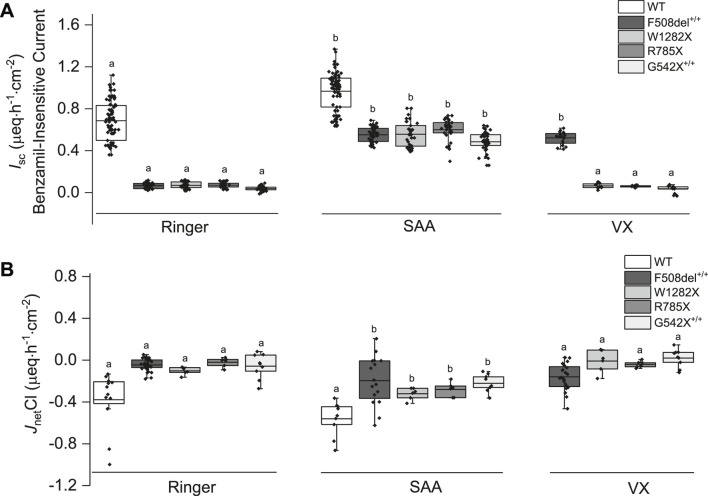
Changes in benzamil-insensitive short-circuit current (*I*_sc_) and corresponding chloride net flux (*J*_net_Cl) in wildtype (WT)- and CF-HBEC with different mutations exposed to Ringer’s solution, the select amino acids (SAA), or a combination of CFTR modulators (VX). **(A)** Benzamil-insensitive *I*_sc_ **(B)** *J*_net_Cl. WT-HBEC, and CF-HBEC with F508del^+/+^, W1282X/R1162X (W1282X), G542X/R785X (R785X), and G542X^+/+^ mutations were treated with DMSO or a combination of 18 µM VX661, 3 µM VX445, and 1 µM VX770 (VX) for 24 h followed by exposure to Ringer’s solution or SAA in Ussing chambers. Three to eight independent experiments were conducted for each donor. The central box represents the interquartile range, with a line inside indicating the mean, and the whiskers representing min and max values. Ringer, SAA, and VX values were compared using one-way ANOVA and *post hoc* Bonferroni tests for pairwise comparisons. Different letters represent significant differences between treatments for each donor (P < 0.05).

Exposure of both WT- and CF-HBEC to SAA in Ussing chambers increased benzamil-insensitive *I*_sc_ and Cl^−^ secretion (Figures 4A,B), and either decreased or did not change benzamil-sensitive *I*_sc_ in CF-HBEC irrespective of the CF mutation, compared to Ringer’s solution.

Benzamil-sensitive *I*_sc_ (electrogenic Na^+^ absorption) was significantly higher in HBEC with all CF mutations (F508del^+/+^: 2.39 ± 0.06 µeq h^−1^ cm^−2^; W1282X/R1162X: 2.62 ± 0.09 µeq h^−1^ cm^−2^; G542^+/+^: 3.10 ± 0.1 µeq h^−1^ cm^−2^) except in G542X/R785X-HBEC where electrogenic Na^+^ absorption decreased (1.82 ± 0.06 µeq h^−1^ cm^−2^; P = 0.002) compared to WT-HBEC (2.14 ± 0.06 µeq h^−1^ cm^−2^). When CF-HBEC were exposed to SAA, benzamil-sensitive *I*_sc_ significantly decreased in G542X/R785X-HBEC (1.66 ± 0.04 µeq h^−1^ cm^−2^, P = 0.033) but remained unchanged in F508del^+/+^ (2.24 ± 0.09 µeq h^−1^ cm^−2^), W1282X/R1162X (2.49 ± 0.08 µeq h^−1^ cm^−2^), and G542^+/+^ (2.95 ± 0.09 µeq h^−1^ cm^−2^) compared to corresponding Ringer controls, suggesting a modest impact of SAA on electrogenic Na^+^ absorption. A 24-h treatment with VX did not change the benzamil-sensitive *I*_sc_.

In the presence of SAA, the benzamil-insensitive *I*_sc_ increased significantly in CF-HBEC compared to their corresponding Ringer values (Figure 4A): F508del^+/+^ (Ringer: 0.07 ± 0.004 µeq h^−1^ cm^−2^ vs. SAA: 0.55 ± 0.01 µeq·h^−1^ cm^−2^), W1282X/R1162X (Ringer: 0.07 ± 0.005 µeq h^−1^ cm^−2^ vs. SAA: 0.56 ± 0.02 µeq h^−1^ cm^−2^), G542X/R785X (Ringer: 0.07 ± 0.005 µeq h^−1^ cm^−2^ vs. SAA: 0.60 ± 0.02 µeq h^−1^ cm^−2^) and G542X^+/+^ (Ringer: 0.04 ± 0.004 µeq h^−1^ cm^−2^ vs. SAA: 0.49 ± 0.01 µeq h^−1^ cm^−2^). Although benzamil-insensitive *I*_sc_ of CF-HBEC exposed to SAA remained lower than that of WT-HBEC exposed to Ringer’s solution (0.68 ± 0.02 µeq h^−1^ cm^−2^), it reached 80.4% in F508del^+/+^, 81.2% in W1282X/R1162X, 87.5% in G542X/R785X, and 70.8% in G542X^+/+^ compared to WT-Ringer controls. In contrast, exposure to VX for 24 h increased benzamil-insensitive *I*_sc_ in Class II mutation (F508del^+/+^) but not in CF-HBEC with Class I mutations compared to CF-HBEC treated with Ringer (Ringer: 0.07 ± 0.004 µeq h^−1^ cm^−2^ vs. VX: 0.52 ± 0.01 µeq h^−1^ cm^−2^; Figure 4A), which represents 84.1% to that of WT-HBEC exposed to Ringer’s solution. The increase in benzamil-insensitive *I*_sc_ with SAA was further confirmed by unidirectional and net ^36^Cl flux studies. *J*_net_Cl showed a significant increase with SAA in CF-HBEC with different mutations suggesting electrogenic Cl^−^ secretion (Figure 4B **;** Table 1).

**TABLE 1:** Basal anion current (benzamil-insensitive current) and corresponding chloride secretion in wildtype and CF-HBEC with different mutations.

Donor	Treatment	*I*_sc_ µeq·h^−1^·cm^−2^	*J*_ms_Cl µeq·h^−1^·cm^−2^	*J*_sm_Cl µeq·h^−1^·cm^−2^	*J*_net_Cl µeq·h^−1^·cm^−2^
Wildtype	Ringer	0.69 ± 0.02	0.93 ± 0.09	1.31 ± 0.09	−0.38 ± 0.08
	SAA	0.97 ± 0.02***	0.92 ± 0.09	1.48 ± 0.11	−0.56 ± 0.06
F508del^+/+^	Ringer	0.07 ± 0.00	0.17 ± 0.01	0.22 ± 0.01	−0.05 ± 0.01
	SAA	0.52 ± 0.01***	0.21 ± 0.03	0.41 ± 0.05	−0.20 ± 0.06*
	VX	0.93 ± 0.09***	0.26 ± 0.02	0.42 ± 0.04	−0.16 ± 0.03
W1282X/R1162X	Ringer	0.07 ± 0.00	0.30 ± 0.01	0.40 ± 0.02	−0.10 ± 0.01
	SAA	0.56 ± 0.02***	0.37 ± 0.01	0.69 ± 0.03	−0.32 ± 0.02**
	VX	0.06 ± 0.01###	0.61 ± 0.01	0.62 ± 0.09	−0.01 ± 0.04###
G542X/R785X	Ringer	0.07 ± 0.00	0.18 ± 0.02	0.20 ± 0.01	−0.02 ± 0.02
	SAA	0.60 ± 0.02***	0.22 ± 0.02	0.50 ± 0.03	−0.28 ± 0.03***
	VX	0.06 ± 0.00###	0.16 ± 0.02	0.20 ± 0.02	−0.04 ± 0.01###
G542X^+/+^	Ringer	0.04 ± 0.00	0.25 ± 0.03	0.31 ± 0.04	−0.06 ± 0.04
	SAA	0.49 ± 0.01***	0.36 ± 0.02	0.58 ± 0.03	−0.22 ± 0.03**
	VX	0.03 ± 0.01###	0.26 ± 0.02	0.24 ± 0.02	0.02 ± 0.03###

Benzamil-insensitive short-circuit current (*I*_sc_) and unidirectional fluxes of ^36^Cl^−^ (*J*_ms_Cl and *J*_sm_Cl) were measured across HBEC, culture inserts under voltage clamp conditions as described in Material and Methods. Wildtype- or CF-HBEC, were treated with DMSO, or a combination of 18 µM VX661, 3 µM VX445, and 1 µM VX770 (VX) for 24 h followed by exposure to Ringer’s solution or the select amino acid combination (SAA) in Ussing chambers. Six to 30 tissue pairs were studied. *J*_ms_Cl, *J*_sm_Cl, and *J*_net_Cl indicate mucosa to serosa, serosa to mucosa, and net fluxes (*J*_ms_ - *J*_sm_), respectively. Values are represented mean ± SEM., One-way ANOVA, followed by *post hoc* Bonferroni was used to compare the treatments (Ringer, SAA, and VX) for each donor.

*P < 0.05, ** P < 0.01, ***P < 0.001 represents significant differences with Ringer, and # P < 0.05, ## P < 0.01, and ### P < 0.001 represents significant differences between SAA, and VX.

### SAA-induced anion current and Cl^−^ secretion are predominantly mediated by SLC26A9

**FIGURE 5:**
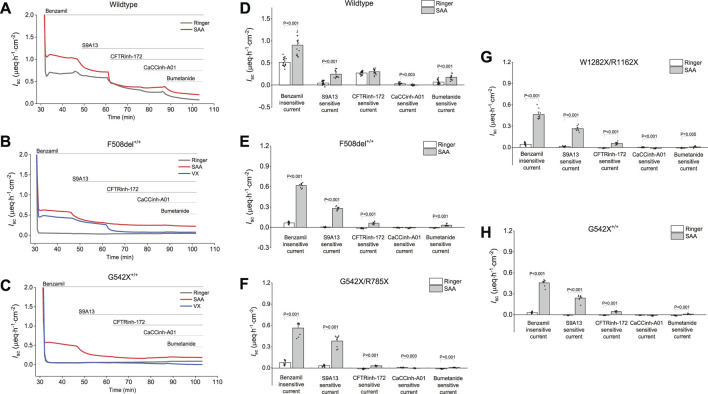
Representative current traces and changes in benzamil-insensitive short-circuit current (*I*_sc_) showing sequential inhibition of short-circuit *I*_sc_ by various anion channel blockers and an NKCC1 inhibitor in wildtype (WT) and CF-HBEC with different mutations in Ussing chambers: **(A)** Representative current traces in wildtype HBEC, and **(B)** CF-HBEC with F508del^+/+^, and **(C)** CF-HBEC with G542X^+/+^ mutations, and **(D)** Changes in benzamil-insensitive *I*_sc_ in wildtype (WT) and **(E)** CF-HBEC with F508del^+/+^, **(F)** CF-HBEC with G542X/R785X, **(G)** CF-HBEC with W1282C/R1162X, and **(H)** CF-HBEC with G542X^+/+^ mutations in Ussing chambers. Cells were treated with DMSO or a combination of CFTR modulators—18 µM VX661, 3 µM VX445, and 1 µM VX770 (VX) for 24 h followed by exposure to Ringer’s solution or the selected amino acid formulation (SAA). After blocking ENaC with benzamil (6 μM; apical), the following inhibitors were added sequentially: 10 µM S9A13 (SLC26A9 inhibitor; apical), 20 µM CFTRinh-172 (CFTR inhibitor; apical and basolateral), 20 µM CaCCinh-A01 (ANO1 inhibitor; apical and basolateral), and 20 µM bumetanide (NKCC1 inhibitor; basolateral). Two to five independent experiments were conducted for each donor. Values are represented as mean ± SEM. Values for Ringer and SAA of each donor were compared using a two-sample t-test. A P-value of 0.05 was considered significant.

The elevated benzamil-insensitive *I*_sc_ induced by SAA in WT- HBEC (Figure 5A), F508del^+/+^-HBEC (Figure 5B) and G542X^+/+^-HBEC (Figure 5C) was sequentially inhibited by various anion channel blockers targeting SLC26A9, CFTR, and ANO1 using S9A13, CFTRinh-172, and CaCCinh-A01, respectively. The portion of the benzamil-insensitive *I*_sc_ not inhibited by the anion channel blockers was further inhibited by an NKCC1 inhibitor (bumetanide), which prevents the basolateral uptake of Cl^−^, essential for its apical exit. Examining the average current changes for WT-, F508del^+/+^-, G542X/R785X, W1282X/R1162X, and G542X^+/+−^HBEC showed that exposure to SAA resulted in a higher S9A13 inhibition in CF-HBEC compared to WT-HBEC (Figures 5D–H). In WT-HBEC, addition of SAA increased S9A13-sensitive *I*_sc_ to 0.25 ± 0.02 µeq·h^−1^·cm^−2^ compared to Ringer (0.05 ± 0.01 µeq·h^−1^·cm^−2^), while CFTRinh-172-sensitive *I*_sc_ was unaltered. The highest S9A13 inhibition was observed in the G542X/R785X mutation (0.38 ± 0.03 µeq·h^−1^·cm^−2^) (Figure 5F), followed by F508del^+/+^ (0.28 ± 0.01 µeq·h^−1^·cm^−2^) (Figure 5E), W1282X/R1162X (0.26 ± 0.01 µeq·h^−1^·cm^−2^) (Figure 5G), and G542X^+/+^ (0.24 ± 0.02 µeq·h^−1^·cm^−2^) (Figure 5H).

To specifically determine the magnitude of CFTR-mediated *I*_sc_ in the presence of SAA, in separate experiments, CFTRinh-172 was used in the absence of any other anion channel blocker, both in the absence (Supplementary Figures S4A,C,E, and S5) or presence of forskolin Supplementary Figures S4B,D,F, and S6). CFTRinh-172-sensitive *I*_sc_ without prior activation of CFTR was significantly lower in WT-HBEC exposed to SAA when compared to Ringer’s solution but was higher in F508del^+/+^-, and G542C^+/+^-HBEC (Supplementary Figures S4A,C,E).

These studies demonstrated that basal SAA-mediated CFTR activity was lower in F508del^+/+^ (P = 0.002), G542X^+/+^ (P < 0.001), and G542X/R785X (P < 0.001) when SLC26A9 was inhibited prior to CFTR inhibition (Figures 5D–H), compared to HBEC without SLC26A9 inhibition (Supplementary Figure S5).

The CaCCinh-A01-sensitive *I*_sc_ was low in both WT-HBEC and CF-HBEC when compared to CFTR- and SLC26A9-mediated anion *I*_sc_ (Figure 5). However, CF-HBEC showed significantly higher CaCCinh-A01-sensitive *I*_sc_ in the presence of SAA when HBEC were not inhibited by S9A13 (Supplementary Figure S5). Similarly, NKCC1-dependent *I*_sc_ was lower with prior addition of S9A13 in SAA-exposed CF-HBEC.

The magnitude of the benzamil-insensitive *I*_sc_ contributed by various anion channels and the remaining NKCC1-mediated *I*_sc_ was not significant between the CF mutations.

Increasing cAMP using the adenylate cyclase activator forskolin increased benzamil-insensitive *I*_sc_ in WT-HBEC but not in CF-HBEC (Supplementary Figures S4B,D,F). The addition of 10 µM forskolin to the apical and basolateral sides in Ussing chambers did not further increase the benzamil-insensitive *I*_sc_ in CF-HBEC exposed to SAA (Supplementary Figure S6).

To investigate the magnitude of Cl^−^ secretion that is mediated by changes in *I*_sc_, *J*_net_Cl was compared to the benzamil-insensitive *I*_sc_ in WT-HBEC and HBEC with Class I and Class II mutations (Table 1). The results showed that in HBEC with F508del^+/+^, 38.5% of the benzamil-insensitive *I*_sc_ was accounted by *J*_net_Cl in the presence of SAA, compared to 17.2% in the presence of VX. In HBEC with Class I mutations, exposure to SAA resulted in a significantly higher ^36^Cl flux compared to VX. In these cells, *J*_net_Cl accounted for 44%–57% of the benzamil-insensitive *I*_sc_. These studies demonstrated that a significant portion of the benzamil-insensitive *I*_sc_ is because of electrogenic Cl^−^ secretion.

In a different set of experiments, we tested whether Cl^−^ secretion (*J*_net_Cl) was responsible for S9A13-sensitive *I*_sc_, in WT-HBEC, and in HBEC with F508del^+/+^ and G542X/F785X mutations (Table 2). The studies demonstrated that the increased S9A13-sensitive *I*_sc_ in the presence of SAA was associated with a significant increase in Cl^−^ secretion in both WT-HBEC and CF-HBEC. The increased Cl^−^ secretion contributed to 72%, 50%, and 39.5% of the S9A13-inhibitable *I*_sc_ in WT-, F508del ^+/+^, and G542X/R785X, respectively. This suggests that a significant portion of S9A13-inhibitable *I*_sc_ is due to electrogenic Cl^−^secretion but could also be partially attributed to HCO_3_^−^ secretion.

**TABLE 2:** S9A13-inhibitable current and corresponding S9A13-inhibitable chloride secretion in wildtype- and CF-HBEC with different mutations.

Donor	Treatment	S9A13-inhibitable *1*_sc_ (µeq·h^−1^·cm^−2^)	Basal *J*_net_Cl µeq·h^−1^·cm^−2^	S9A13-inhibitable *J*_net_Cl (µeq·h^−1^·cm^−2^)
Wildtype	Ringer	0.05 ± 0.01	−0.24 ± 0.04	0.13 ± 0.08
	SAA	0.25 ± 0.02***	−0.58 ± 0.06**	−0.18 ± 0.07#
F508del^+/+^	Ringer	0.01 ± 0.00	−0.05 ± 0.02	−0.04 ± 0.04
	SAA	0.28 ± 0.01***	−0.23 ± 0.02*	−0.14 ± 0.04
G542X/R785X	Ringer	0.03 ± 0.00	−0.03 ± 0.02	−0.02 ± 0.03
	SAA	0.38 ± 0.02***	−0.24 ± 0.02***	−0.15 ± 0.03##

S9A13-inhibitable short-circuit current (*I*_sc_) and unidirectional fluxes of ^36^Cl^−^ were measured across the HBEC, culture inserts under voltage clamp conditions as described in Material and Methods. Wildtype- or CF-HBEC, were treated with DMSO, for 24 h followed by exposure to Ringer’s solution or the select amino acid combination (SAA) in Ussing chambers. Three to 9 tissue pairs were studied. The net Cl^−^ flux (*J*_net_Cl) was calculated by subtracting serosa to mucosa from mucosa to serosa flux values. Values are represented as mean ± SEM., Two-sample t-test was used to compare the treatments (Ringer, SAA) for each donor.

*P < 0.05, ** P < 0.01, ***P < 0.001 represents significant differences with Ringer. # P < 0.05, and ## P < 0.01 represents significant differences between *I*_sc_ and *J*_net_Cl.

These findings indicate that SAA increased benzamil-insensitive *I*_sc_ by electrogenic Cl^−^ secretion in both Class I and Class II mutations, but electrogenic HCO_3_^−^ secretion may have contributed to the higher anion *I*_sc_. The majority of SAA-mediated *I*_sc_ and *J*_net_Cl changes were attributed to increased SLC26A9 activity. The level of anion secretion achieved was comparable to that in normal WT-HBEC. In addition, the SAA-stimulated benzamil-insensitive *I*_sc_ exhibited significantly higher electrogenic Cl-secretion when compared to treatment with VX in F508del^+/+^, though the total benzamil-insensitive *I*_sc_ was higher in the presence of VX.

### Whole-cell analysis showed SAA-mediated anion current in HBEC with class I and class II mutations

**FIGURE 6:**
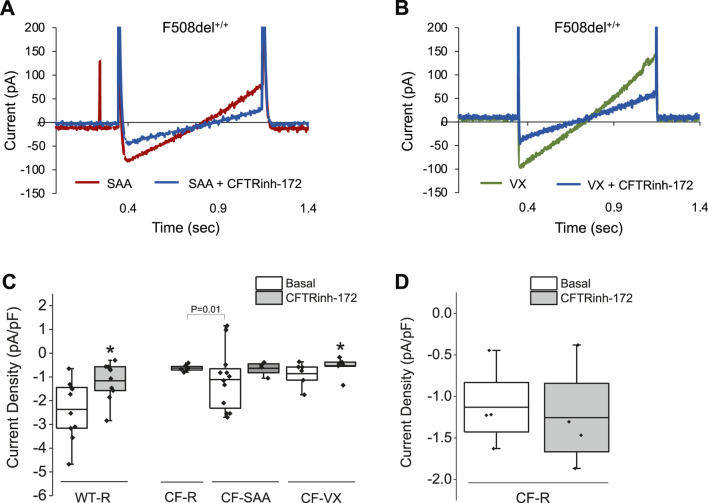
Representative whole-cell recordings for current density in response to 10 µM CFTRinh-172 in wildtype (WT)- and CF-HBEC (F508del^+/+^). Cells were treated with DMSO or a combination of 18 µM VX661, 3 µM VX445, and 1 µM VX770 (VX) for 24 h followed by exposure to Ringer’s solution (R) or the select amino acid combination (SAA). **(A)** Representative current density responses in F508del^+/+^-HBEC exposed to SAA or SAA containing CFTRinh-172. **(B)** Representative current density responses in VX-treated F508del^+/+^-HBEC exposed to Ringer’s solution or Ringer containing CFTRinh-172. **(C)** WT- and CF-HBEC treated with DMSO or VX for 24 h followed by exposure to Ringer’s solution (WT-R, CF-R) or SAA (CF-SAA). Changes in current density were recorded at 10 mV from 15-min baseline recordings (Basal), after the Ringer’s solution was replaced by SAA (CF-AA), and after the addition of CFTRinh-172. **(D)** In a different set of experiments, changes in current density were plotted in response to CFTRinh-172 in CF-HBEC exposed to Ringer’s solution. The current density was determined from current differences before and after blocking CFTR with CFTRinh-172. Currents were normalized for cell capacitance (n = 4 to 8 cells). The central box represents the interquartile range, with a line inside indicating the mean, and the whiskers representing minimum and maximum values. Baseline values were compared to values after CFTRinh-172 addition using a paired t-test (*P < 0.05). Statistical differences between CF-R and CF-SAA were assessed using a two-sample t-test. A P-value of 0.05 was considered significant.

**FIGURE 7:**
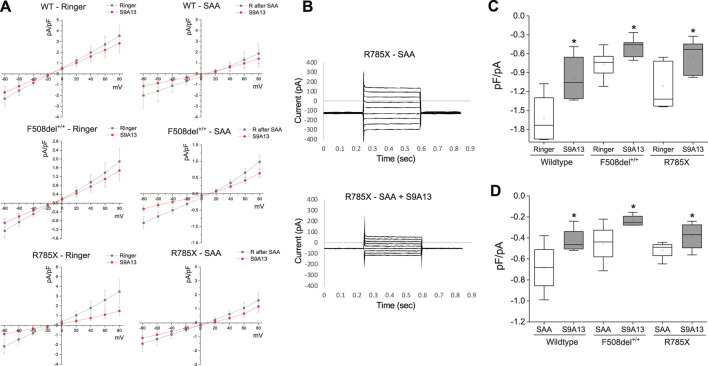
Select amino acid (SAA)-evoked currents in Wildtype (WT)- and CF-HBEC with F508del^+/+^ and G542X/R785X (R785X) mutations. **(A)** Representative current-voltage (IV) relationships were recorded from WT-, and CF-HBEC when exposed to Ringer’s (R) solution or SAA using the whole-cell patch-clamp technique. Cells were held at a baseline holding potential of −10 mV and subjected to voltage steps ranging from −80 to +80 mV for several minutes at 3-min intervals to prevent accumulation of voltage-dependent effects. **(B)** Voltage step protocols in R785X-HBEC when exposed to SAA, and SAA containing S9A13 (SLC26A9 inhibitor). Current responses were measured and plotted as a function of the applied voltage to generate IV curves. Traces show the average current amplitude across the range of test potentials. **(C, D)** Current responses to S9A13 in WT- and CF-HBEC exposed (n = 5 to 6 cells) to **(C)** HBEC exposed to Ringer’s solution or Ringer’s solution containing S9A13, and **(D)** HBEC exposed to SAA or SAA containing S9A13. The central box represents the interquartile range, with a line inside indicating the mean, and the whiskers representing min and max values. Ringer and SAA values were compared to values after adding S9A13 using a paired t-test (*P < 0.05). A P-value of 0.05 was considered significant.

The study thus far has shown the effectiveness of the SAA in stimulating electrogenic Cl^−^ secretion and potential HCO_3_^−^ secretion. Whole-cell patch clamp recordings were used to study ion channel activity, and the measurements showed that SAA or VX increased current density, which could be blocked by CFTRinh-172 (Figure 6) or S9A13 (Figure 7) suggesting inhibition of Cl^−^ secretion. Class II HBEC (F508del^+/+^) were treated with VX or DMSO for 24 h and then exposed to Ringer’s solution or SAA for 15 min during the recordings. Representative current traces in CF-HBEC exposed to SAA or treated with VX before and after the addition of CFTRinh-172 are shown in Figures 6A,B. Exposure of WT-HBEC to CFTRinh-172 resulted in a significant reduction in current density (−2.37 ± 0.39 pA/pF vs. −1.16 ± 0.25 pA/pF, n = 10, P < 0.05; Figure 6C). When CF-HBEC (F508del^+/+^) were exposed to SAA, current density significantly increased from −0.63 ± 0.04 pA/pF to −1.51 ± 0.29 pA/pF (n = 8; P = 0.01; Figure 6C). Total current density decreased in the presence of CFTRinh-172 in SAA- or VX-treated cells, from −1.38 ± 0.39 to −0.64 ± 0.15 pA/pF (n = 4; P = 0.06), and from −0.86 ± 0.21 to −0.55 ± 0.17 pA/pF (n = 6; P = 0.02), respectively, representing CFTR-mediated currents (Figure 6C). In a separate series of experiments, F508del^+/+^ -HBEC exhibited no detectable CFTR-activity when exposed to Ringer’s solution (−1.13 ± 0.25 pA/pF vs. −1.26 ± 0.32 pA/pF, n = 4; Figure 6D). These results are consistent with an increase in CFTR function as demonstrated in SAA-exposed CF-HBEC in Ussing chamber experiments.

To test the efficacy of the S9A13 blocker and the amount of SLC26A9-carried current in WT-, F508del ^+/+^- and G542X/R785X-HBEC, a series of IV protocols were run for several minutes with an interval of 3 min before and after adding the blocker (Figures 7A,B). Adding S9A13 showed a significant change in SLC26A9-carried current in WT-, F508del^+/+^- and G542X/R785X-HBEC perfused with SAA compared to Ringer’s solution (Figures 7C,D). These experiments were repeated on cells perfused with SAA for 15 min and washed with Ringer’s solution for another 15 min before adding the blocker.

In addition, IV protocols performed in G542X/R785X were maintained at a holding potential of minus 20 mV for a total recording of up to 70 min. These experiments were performed in the presence of Ringer’s solution, SAA, and both SAA and the S9A13 blocker. The amount of current was compared at minus 15–20 *versus* 50–60 mV to highlight a percentage change in current. In G542X/R785X-HBEC, Ringer’s solution showed a 95.6% ± 6% change (n = 6; N.S.); exposure to SAA showed a 203.6% ± 37.6% change, (n = 5; P = 0.045); and treatment with SAA and S9A13 showed a 63.8% ± 9.6% change (n = 5; P = 0.006).

### Q-PCR analysis showed increased mRNA expression of anion channels with exposure to SAA in HBEC with class I and class II mutations

**FIGURE 8:**
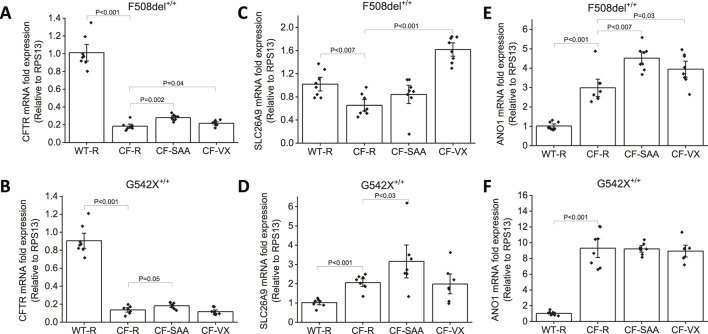
Changes in CFTR, SLC26A9, and ANO1 mRNA levels in Wildtype (WT)- and CF-HBEC with F508del^+/+^-, and G542X^+/+^ mutations and various treatments. Cells were treated with DMSO or a combination of 18 µM VX661, 3 µM VX445, and 1 µM VX770 (VX) for 24 h followed by a 4-h exposure to Ringer’s solution (R) or the select amino acid combination (SAA). Values were represented as fold change with respect to RPS13, and normalized to WT-HBEC. **(A)** CFTR mRNA levels in F508del^+/+^-HBEC, **(B)** CFTR mRNA levels in G542X^+/+^-HBEC, **(C)** SLC26A9 mRNA levels in F508del^+/+^-HBEC, **(D)** SLC26A9 mRNA levels in G542X^+/+^-HBEC, **(E)** ANO1 mRNA levels in F508del^+/+^-HBEC, **(F)** ANO1 mRNA in G542X^+/+^-HBEC. Four independent experiments were conducted for each donor. Values are represented as mean ± SEM. The Kruskal–Wallis test was used for the overall comparison of mRNA levels, and the Mann-Whitney U-test was used for pair-wise comparison. A P-value of <0.05 was considered significant.

The mRNA expression levels of anion channels such as CFTR, SLC26A9, and ANO1 were measured in CF-HBEC with homozygous F508del^+/+^ and G542X^+/+^ mutations exposed to SAA or VX (Figures 8A–F). CFTR mRNA levels (Figures 8A,B) were reduced in CF-HBEC compared to WT-HBEC (WT: 1.01 ± 0.06; F508del^+/+^: 0.18 ± 0.02; P < 0.001) in Ringer’s solution. However, apical exposure to SAA or treatment with VX increased CFTR mRNA levels compared to untreated CF controls (SAA: 0.28 ± 0.01, P = 0.002; VX: 0.22 ± 0.01, P = 0.04 vs. 0.18 ± 0.02). In G542X^+/+^, CFTR mRNA levels were significantly lower compared to WT-HBEC (WT: 0.90 ± 0.06, G542X: 0.14 ± 0.02; P < 0.001; Figure 8B). Addition of SAA to the apical side of CF-HBEC showed a small but significant increase in CFTR mRNA levels (0.18 ± 0.01; P = 0.05). However, in VX-treated G542X^+/+^-HBEC, mRNA levels remained unchanged (Figures 8A,B). These studies suggest the activation of a potential read-through mechanism by SAA.

The mRNA expression of SLC26A9 (Figure 8C) was lower in HBEC with the F508del^+/+^ mutation compared to WT-HBEC (F508del^+/+^: 0.65 ± 0.07; WT: 1.02 ± 0.08, P < 0.007). Treatment with VX significantly increased SLC26A9 mRNA levels and such an increase was not seen with exposure to SAA in HBEC with F508del^+/+^ (VX: 1.62 ± 0.08, P < 0.001). SLC26A9 mRNA expression levels in G542X^+/+^ were significantly higher compared to WT-HBEC (WT: 1.02 ± 0.07; G542X ^+/+^: 2.06 ± 0.13; P < 0.001). Exposure to SAA showed a 3.16-fold increase in SLC26A9 mRNA levels when compared to WT-HBEC, and this increase was significantly higher than in G542X ^+/+^-HBEC exposed to Ringer’s solution (3.16 ± 0.57, P < 0.03). VX-treated G542X ^+/+^-HBEC did not show a similar increase in SLC26A9 mRNA (Figure 8D).

In contrast, ANO1 mRNA levels (Figures 8E,F) were increased in both F508del^+/+^-, and G542X^+/+^-HBEC when compared to WT-HBEC (WT: 1.02 ± 0.08; F508del^+/+^: 2.98 ± 0.29, P < 0.001; G542X^+/+^: 9.31 ± 0.80, P < 0.001). Treatment with VX and exposure to SAA further increased ANO1 mRNA levels in F508del-HBEC (VX: 3.94 ± 0.27, P = 0.03; SAA: 4.51 ± 0.22, P < 0.007), but such an increase was not observed in G542X^+/+^-HBEC.

### Immunofluorescence imaging showed increased anion channel protein expression with exposure to SAA in HBEC with class I and class II mutations

**FIGURE 9:**
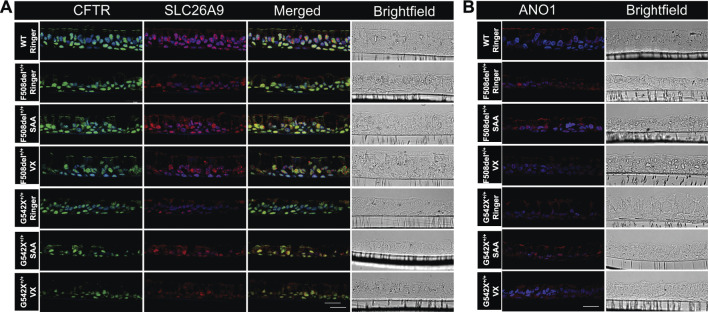
Immunofluorescence imaging for **(A)** CFTR, and SLC26A9, and **(B)** ANO1 protein in wildtype (WT)-HBEC, and CF-HBEC with F508del^+/+^-, and G542X^+/+^ mutations treated with DMSO or a combination of 18 µM VX661, 3 µM VX445, and 1 µM VX770 (VX) for 24 h followed by a 1-h exposure to Ringer’s solution or the select amino acid combination (SAA). CFTR is shown as a green signal (Alexa Fluor 488), and SLC26A9 and ANO1 are shown as a red signal (Alexa Fluor 647). DAPI was used for nuclei stain (blue signal). The scale bar corresponds to 20 µm. All experiments were performed on three different filters per donor.

Immunofluorescence imaging for CFTR and SLC26A9 protein expression in WT-HBEC cultures showed a strong signal along the apical membranes of both ciliated and non-ciliated cells (Figure 9A; Supplementary Figure S7). Considering the low abundance of ionocytes in bronchial epithelium (<1%), we did not specifically differentiate this cell type from other non-ciliated cells by immunofluorescence labeling. However, cell morphology and localization of single, positively labeled cells indicate the presence of CFTR in this cell type. CFTR-positive signals were also present within or near nuclei, a phenomenon previously described due to CFTR protein expressed within the endoplasmic reticulum, due to non-specific staining of nuclear protein, or contaminating mouse Ig when using the monoclonal mouse antibody clone 596 (van Meegen et al., 2011; Lukasiak and Zajac, 2021; Sato et al., 2021). SLC26A9 was co-expressed with CFTR but of a lower intensity. Additionally, SLC26A9 was independently present within tight junctions, separate from CFTR (Figure 9A). In CF-HBEC, CFTR membrane expression was significantly reduced in F508del^+/+^-HBEC and absent in G542X^+/+^-HBEC, although some SLC26A9 signals remained visible at the apical membrane. Exposure to SAA in HBEC with Class I or II mutations resulted in notably higher CFTR and SLC26A9 expression at the apical membrane. Additionally, increased SLC26A9 signals were observed independently of CFTR at the apical membrane. VX treatment enhanced CFTR membrane expression in HBEC with F508del^+/+^ but showed no CFTR signal in G542X^+/+^. These findings suggest that SAA elevates CFTR and SLC26A9 protein expression at the apical membrane of CF-HBEC and may promote new synthesis of both proteins, regardless of the mutation type. In WT-HBEC, ANO1 protein was abundantly expressed along the apical membrane of both ciliated and non-ciliated cells (Figure 9B). Exposure to SAA, but not VX treatment, increased ANO1 protein levels in HBEC with Class I and II mutations. Further studies are necessary to determine the significance of the increased ANO1 protein levels in CF-HBEC, as its expression along the apical membrane was reduced in immunofluorescence images. However, the CaCCinh-A01-sensitive *I*_sc_ showed a small but significant increase with SAA treatment in all CF mutations.

### SAA enhanced airway surface liquid, ciliary beat frequency, and mucociliary motility in CF-HBEC

**FIGURE 10:**
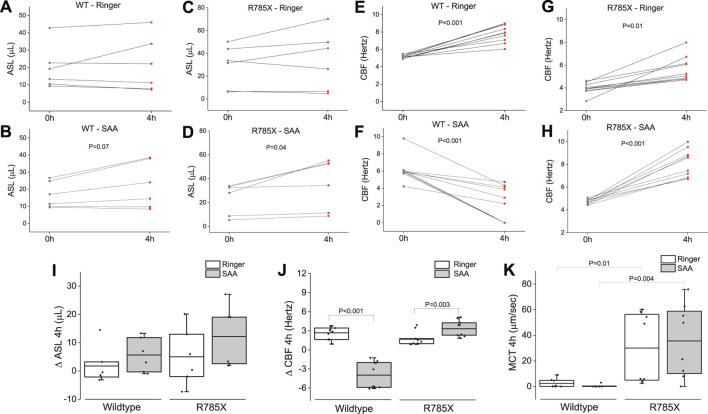
Airway surface liquid (ASL) volume, ciliary beat frequency (CBF), and mucociliary transport (MCT) in wildtype (WT)-, and G542X/R785X (R785X)-HBEC exposed to Ringer’s solution or the select amino acid combination (SAA) measured at timepoint zero and 4 h after exposure. **(A, B)** Individual changes of ASL in WT-HBEC exposed to Ringer’s solution or SAA, **(C, D)** Individual changes of ASL in R785X-HBEC exposed to Ringer’s solution or SAA, **(E, F)** Individual changes of CBF in WT-HBEC exposed to Ringer’s solution or SAA, **(G, H)** Individual changes of CBF in R785X-HBEC exposed to Ringer’s solution or SAA. **(I)** Comparison of ASL volumes (∆ delta changes for 4 h) in wildtype- and R785X-HBEC exposed to Ringer’s solution or SAA. **(J)** Comparison of CBF (∆ delta changes for 4 h) in wildtype-, and R785X-HBEC exposed to Ringer’s solution or SAA. **(K)** Comparison of MCT in wildtype-, and R785X-HBEC after 4 h of exposure to Ringer’s solution or SAA. The central box represents the interquartile range, with a line inside indicating the mean, and the whiskers representing minimum and maximum values. Three to four independent experiments were conducted for each donor. Values at time point zero and after 4 h were compared using a paired t-test. A two-sample t-test was used to compare the treatment groups (Ringer and SAA). A P-value of <0.05 was considered significant.

Airway surface liquid, CBF, and MCT are critical for clearing inhaled particles and pathogens from the airways—functions that are compromised in individuals with CF. To simulate inhalation therapy, we nebulized either Ringer’s solution or SAA onto the apical surface of wildtype- and Class I CF-HBEC (G542X/R785X). Four hours post-exposure, ASL levels were elevated in both WT- and CF-HBEC exposed to SAA compared to those exposed to Ringer’s solution (Figures 10A–D). A similar trend was observed in CBF, which also increased in CF-HBEC following SAA exposure (Figures 10E–H). Summary data for ASL and CBF responses indicate that, while ASL was higher in SAA-treated WT-HBEC than in those exposed to Ringer (Figure 10I), CBF was reduced in WT-HBEC following SAA exposure yet increased in CF-HBEC under the same conditions (Figure 10J). Additionally, MCT was higher in CF-HBEC exposed to either Ringer or SAA compared to WT-HBEC under both treatment conditions (Figure 10K). These results contrast with the well-established mucociliary dysfunction typically seen in CF and warrant further investigation. Enhancing ASL, CBF, and MCT could offer significant therapeutic benefits and improve quality of life for patients with CF.

## Discussion

In this study, VX and forskolin treatment rescued 69.8% of the CFTR function in F508del^+/+^-HBEC compared to WT-HBEC, aligning with previous findings (Veit et al., 2020). However, CF-HBEC with heterozygous or homozygous nonsense mutations showed less or no improvement in CFTR function. Sequentially inhibition of this current using anion channel blockers targeting SLC26A9, CFTR, and ANO1, suggested potential role for SLC26A9, CFTR and ANO1. CFTR inhibitor 172-sensitive portion of the SAA-induced anion *I*_sc_ (benzamil-insensitive) in F508del^+/+^-HBEC and G542X^+/+^-HBEC was ∼10%. qPCR analysis showed a modest increase in CFTR mRNA levels, while immunofluorescence indicated higher CFTR expression along the HBEC apical region. These findings potentially suggest increased read-through and rescue of the CFTR function. Roy et al. found that the insertion of individual AAs was distinct for specific nonsense codons and read-through-inducing agents (Roy et al., 2015).

It was identified that glutamine, tyrosine and lysine are inserted at UAA and UAG stop codons, whereas tryptophan, arginine, and cysteine are inserted at UGA in an *in vitro* yeast model (Roy et al., 2015). Interestingly, G542X mutation results in an in-frame opal (UGA) termination codon at glycine 542 of CFTR, making cysteine a potential candidate for read-though at this PTC. Based on current data, the AAs in SAA such cysteine, lysine or tyrosine could promote the generation of near-cognate tRNAs to facilitate read-through at the PTC. Increased cognate AA-tRNA incorporation mechanism for increased mRNA for anion channels other than CFTR is unlikely as the qPCR showed decreased SLC26A9 mRNA in the presence of SAA. Although the SAA-induced benzamil-insensitive portion of the ANO1-mediated *I*_sc_ was relatively low, increased transcription and translation are possible with ANO1, as both mRNA and protein expression levels were increased. However, ANO1 expression along the apical membrane of CF-HBEC was low and could explain the reduced functional activity. The fate of the increased ANO1 protein levels and its expression with SAA needs to be further explored. It is thus possible that the major portion of SAA-induced anion secretion observed in F508del^+/+^-HBEC (45%) and G542X^+/+^-HBEC (52%) could be partially resulting from increased read-through. Alternatively, SLC26A9 protein expression levels could be directly influenced by SAA.

The main route for AA signal transduction are intra- or extracellular binding proteins, such as transporters, receptors, enzymes, or nucleic acids that activate signaling pathways, and control protein synthesis or regulate protein activities such as opening or closing of ion channels (Hyde et al., 2003; Broer and Broer, 2017). Here, we investigated the unique potential of AAs to modulate plasma membrane expression and activity of distinct channels and transporters that facilitate transepithelial anion secretion and electrogenic Na^+^ absorption in airways. We demonstrated that a set of five select AAs (SAA) can increase the expression levels and activities of CFTR, ANO1, and SLC26A9 using an *in vitro* HBEC culture model to study CF with Class I and Class II mutations. We could show that SAA restores net Cl^−^ secretion in primary CF-HBEC with two of the most common mutations (Class I and II) to a level that is comparable to the secretory capacity of normal WT-HBEC. The anion *I*_sc_ in the presence of SAA was partially mediated by increased CFTR mRNA levels, increased protein expression and enhanced channel activity at the apical membrane, and the activation of alternative anion secretory pathways such as ANO1 and SLC26A9.

Additionally, some read-through agents have been commonly used in CF patients, but clinical safety and limited bioavailability of those compounds at the cellular level are still an issue, and applicable read-through therapeutics for CF nonsense mutations are still in their developmental stage. To our surprise, SAA activated a marked CFTR-mediated *I*_sc_ in HBEC with class I mutations that are usually characterized by a lack of CFTR protein or minimal synthesis of a truncated protein version. Endogenous tRNA carrying distinct AAs have been shown to function as suppressor tRNAs in prokaryotic cells, with tRNA carrying tryptophan, cysteine, or arginine to misread the third or first base of UGA-PTC, while read-through of UAG and UAA inserts glutamine, tyrosine or lysine (Pibiri et al., 2020). Increased expression of tryptophan and tyrosine tRNAs elevate stop codon read-through in human cell lines (Beznosková et al., 2021). The amount of rescued protein by natural read-through is generally very small, however, the frequency of read-through at PTCs is about 10-fold higher (0.01%–1%) (Lombardi et al., 2020). The discovery of suppressor tRNAs, combined with the development of methods to chemically aminoacylate these tRNAs with non-cognate AAs was a major breakthrough in drug development (Hendrickson, 2003). More than 100 structurally diverse AAs have been incorporated into proteins using this method, demonstrating that once a tRNA is aminoacylated, the translation apparatus is reasonably promiscuous for different AAs.

Alternate secretory pathways, especially calcium-activated anion secretion and SLC26A9-mediated transport mechanisms, are of great interest in the CF field. Members of the SLC26 family have been functionally linked to CFTR activation (Dorwart et al., 2007). Some SLC26A family members have PDZ-domain-interacting motifs that allow association with CFTR via scaffolding proteins. These CFTR-SLC26A complexes likely associate with cytoskeleton-interacting proteins (e.g., ezrin) and regulatory proteins such as PKA (Sun et al., 2000). Studies by Ko et al. (2004) *suggest mutual stimulatory interactions between SLC26A3, ANO1, and CFTR involve the R region of CFTR and the STAS domain of A6, potentially influencing CFTR gating.* This may explain how PKA-induced CFTR stimulation can generate tissue-specific secretory products, and diverse transport phenotypes when CFTR is absent or impaired, as in CF (Frizzell and Hanrahan, 2012). The identification and functional characterization of lung-specific SLC26A9 in 2002 (Lohi et al., 2002) suggested a mechanism for airway anion secretion linked CFTR activity. Bertrand et al. (2017) found SLC26A9 is constitutively active without stimulating agents and significantly contributes to transepithelial anion *I*_sc_ in airways. SLC26A9 is inhibited by the CFTR pore blocker GlyH-101, and appears to require functional CFTR to maintain its basic activity at the plasma membrane (Bertrand et al., 2009). When SLC26A9 is co-expressed with F508del CFTR, defective CFTR trafficking leads to retention of SLC26A9, indicating potential SLC26A9 downregulation in CF (Bertrand et al., 2017). This could explain the reduced protein expression levels of SLC26A9 observed in HBEC with F508del^+/+^ and Class I mutation exposed to Ringer’s solution or SAA. However, HBEC with F508del^+/+^ and Class I mutation treated with SAA exhibited S9A13-sensitive current, constituting 45.3% and 52.2% of the total benzamil-insensitive *I*_sc_ respectively, suggesting that SLC26A9 can function even with defective or absent CFTR. Alternatively, it is possible that SAA treatment in CF-HBEC increased read-through, producing a small amount of functional CFTR protein, which can co-function with the existing SLC26A9.

^36^Cl flux studies showed that S9A13 inhibited ∼60% of the basal *J*_net_Cl in the presence of SAA in F508del ^+/+^- or G542X/R785X-HBEC. The incomplete inhibition suggests reduced specificity of the blocker to SLC26A9, inadequate blocker concentration, or possible involvement of electrogenic HCO_3_^−^ secretion or electrogenic cation absorption not inhibited by S9A13 (Kunzelmann et al., 2023). Studies have indicated a potential HCO_3_^−^ secretion via SLC26A9. Further studies are necessary to determine the magnitude of the HCO_3_^−^ secretion if any.

A second alternative anion-secretory pathway is the apical calcium-activated Cl^−^ channel anoctamin 1 (ANO1). ANO1 protein is a dimer with each subunit containing an ion conduction pore that mediates anion-selective *I*_sc_ following an increase in intracellular Ca^2+^. It has been shown that ANO1 is essential for proper activation and membrane expression of CFTR, resulting in a functional overlap of cAMP- and Ca^2+^-dependent Cl^−^ transport (Ousingsawat et al., 2011; Benedetto et al., 2017). In the presence of SAA, the CF-HBEC (Class I and Class II mutation) exhibited a small but significant CaCCinh-A01-sensitive *I*_sc_. qPCR showed increased mRNA levels, and immunofluorescence showed increased protein expression levels, in SAA-treated CF-HBEC. Such changes were not observed with VX treatment. The increased CFTR activity observed in both Class I and Class II mutations could result from the overlap of cAMP- and Ca^2+^-dependent Cl^−^ transport. Future studies are essential to determine the role of ANO1 in increased CFTR and SLC26A9 activity with SAA treatment.

The precise mechanism by which AAs in SAA enter the cell and/or activate the signaling events in WT- and CF-HBEC, leading to anion transport system activation, remains unclear. However, saturation kinetic studies using SAA suggest a potential role for AA transporters. AA-mediated cell signaling can occur either directly through the AA transporter via substrate binding or indirectly through secondary changes associated with AA transport, or through substrate-triggered responses via extracellular or intracellular receptors (Hyde et al., 2003). AA transport systems are classified based on substrate specificity, transport mechanisms (Christensen, 1990), or genetic similarities within the solute carrier (SLC) family (Hediger et al., 2013). Studies have shown that in airways, secreted proteins and peptides from mucins, lysozyme, transferrin, defensins, and surfactant must be degraded by proteases and peptidases that are present within the airways, allowing single AAs to cross the apical membrane through transporters (Folkesson et al., 1996). Various AA transporters that are Na^+^- and Cl^−^-dependent, and Na^+^-independent and neutral AAs transporters have been recognized in airway epithelium at the basal or apical membrane (Knickelbein et al., 1997; Galietta et al., 1998; Sloan et al., 2003; Kowalczuk, et al., 2005; Rotoli et al., 2005; Kanai et al., 2013; Rotoli et al., 2020). Given that intracellular AA concentrations are generally higher than extracellular levels, AA transport against the electrochemical gradient is often coupled with cotransport of Na^+^, H^+^, Cl^−^ or counter-transport of K^+^ (Bröer, 2002). Analyzing AA transport activities for specific cell types is challenging due to overlapping substrate specificities, varying affinities, and unclear subcellular localization (Gauthier-Coles et al., 2021). However, further studies in these areas will help elucidate the mechanisms by which SAA activates anion secretory activity.

SAA’s anion-secretory characteristics could be based on the mode of action of five AAs with at least three different transport stoichiometries that contribute to the overall changes in *I*_sc_ and Cl^−^ secretion. Proline, glycine, and cysteine stimulated most of the anion *I*_sc_ via low-affinity transport systems with proline mainly activating ANO1 and other alternative channels or transporters, while cysteine increased CFTR-mediated and alternative anion secretion, and glycine stimulated all three anion-secretory pathways. Even though the high-affinity transporters for lysine, cysteine, and glycine increased basal *I*_sc_ by around 0.2 µeq·h^−1^·cm^−2^, the increase in *I*_sc_ was not associated with activation of CFTR, ANO1, or NKCC1-mediated transport systems. Saturation kinetics in Ringer’s solution and modified Ringer’s solution with reduced Na^+^- and Cl^−^, showed that the high-affinity transporter for glycine and cysteine was strictly Na^+^-dependent and non-functioning in the absence or at low concentrations of Na^+^ while lysine’s high-affinity transporter was also negatively affected by lack of Na^+^ suggesting that the three AAs are most likely transported by the same transporter at low concentrations (Shi et al., 2021).

The effect of SAA on benzamil-insensitive *I*_sc_ and Cl^−^ secretion in CF-HBEC may result from a complex interplay of AA transporters and apical ion availability. While the exact mechanisms remain speculative, glycine, proline, and cysteine among the five AAs in SAA mediated most of the increased anion *I*_sc_. These AAs are unique in their multifaceted effects on protein structure and function, making them ideal candidates for modulating the synthesis, maturation, trafficking, and activity of membrane proteins. Glycine, a constituent of glutathione, plays a crucial role in anti-oxidative homeostasis in intestinal epithelial cells. It can trigger Cl^−^ influx via ionotropic receptors, resulting in increased Cl^−^ conductance and hyperpolarization at synapses in the spinal cord, Kupffer cells and white blood cells (Wheeler M. et al., 2000; Wheeler M. D. et al., 2000). Proline functions as a cytoplasmic radical scavenger by quenching hydroxyl radicals and stabilizes proteins in their natural conformation, preventing protein denaturation and accumulation of misfolded proteins. At high concentrations, proline is used to charge tRNA molecules (Patriarca et al., 2021). Cysteine is a highly conserved AA residues in proteins, responsible for diverse functions, including regulation of catalysis, structure, redox sensitivity, and metal trafficking (Bak et al., 2019). Due to its highly ionizable and oxidation-sensitive sulfur atom, cysteine plays a key role in the cellular redox system. It serves as an antioxidant via glutathione thereby modulating posttranslational modifications of regulatory proteins and generates intracellular secondary messengers (Paulsen and Carroll, 2013). In the ER, cysteine supports protein folding and trafficking by maintaining structural and functional integrity of secreted proteins through disulfide-bond formation (Bak et al., 2019). Proline and glycine with their physico-chemical properties, affect the geometry of transmembrane domains and protein trafficking in cells (Glatzova et al., 2021). Proline and glycine are defined as typical ‘helix-breakers’ (Levitt, 1978). An oxidizing environment and disulfide bonds favor interactions required for full-size CFTR openings, while reducing conditions allow greater sub-conductance openings. The complex properties of proline, glycine and cysteine make them key modulators of posttranslational modifications, trafficking and membrane stability, together with the potential ability of cysteine, lysine and tyrosine to function as read-through agents seem to be the key elements for the mechanisms of action of SAA in CF-HBEC. Notably, SAA may also reduce sodium absorption through ENaC (benzamil-sensitive current); however, the results were inconsistent and highly donor-specific, and further research is required to clarify their effect on ENaC-mediated sodium absorption.

We demonstrate that a specific set of amino acids (SAA) can activate and enhance the expression and function of CFTR, ANO1, and SLC26A9 mRNA in CF-HBEC with various mutations. SAA increased Cl^−^ and/or HCO_3_^−^ secretion, followed by fluid secretion, as confirmed by measuring ASL. SAA administration on to the apical surface Class I G542X/R785X-HBEC increased ASL, CBF, and MCT suggesting that SAA treatment may help restore key airway defense mechanisms by improving mucus hydration and mucociliary clearance, typically compromised in cystic fibrosis.

The enhancement of crucial airway clearance mechanisms through increased Cl^−^ secretion via elevated SLC26A9 and CFTR expression and function underscores SAA’s potential to combat CF-related impairments in airway function. By restoring these essential processes, SAA may significantly reduce the risk of infections and improve lung function, ultimately promoting better respiratory health for individuals with cystic fibrosis. However, AA availability *in vivo* may differ from *in vitro* conditions, and identifying the most effective delivery method remains to be studied. Previous studies have shown efficacy of AA formulations both *in vitro* and *in vivo*, oral administration of the formulations was recommended during the inter-digestive phase to optimize efficacy (Chitwood et al., 2022; Chauhan et al., 2021). To ensure direct tissue exposure and maximize therapeutic impact, we envision delivering the formulation via aerosol or by nebulization.

This suggests that SAA may modulate critical processes such as protein translation, trafficking, and membrane activity, offering a potential therapeutic avenue to improve ion transport and function in CF patients, regardless of their specific mutations.

## Data Availability

The raw data supporting the conclusions of this article will be made available by the authors, without undue reservation.
