# Supplementary material for: Selective amino acid formulation enhances anion secretion and restores function in cystic fibrosis mutations
Source: Front Pharmacol. 2025 Aug 4;16:1522130. doi: 10.3389/fphar.2025.1522130 (PMC12358407; doi:10.3389/fphar.2025.1522130)
Supplement: Supplementary file 1 [file Supplementaryfile1.docx]

**Supplementary Figure Legends**

**Suppl. Figure 1**: Representative rankings of individual amino acids (8 mM, added to both apical and basolateral sides) based on their effects on changes in short-circuit current (*I*_sc_) in response to benzamil (ENaC inhibition; 6 µM; apical), CFTRinh-172 (CFTR inhibition; 20 µM; apical and basolateral), CaCCinh-A01 (ANO1 inhibition; 10 µM; apical and basolateral), and bumetanide (NKCC1 inhibition; 20 µM; basolateral) in CF-HBEC with the G542X^+/+^ mutation: **(A)** Benzamil-sensitive *I*_sc_, **(B)** Benzamil-insensitive *I*_sc_, **(C)** CFTRinh-172-sensitive *I*_sc_, **(D)** CaCCinh-A01-sensitive *I*_sc_, and **(E)** Bumetanide-sensitive *I*_sc_. Two independent experiments were conducted for each amino acid.

**Suppl. Figure 2:** Representative saturation kinetic plots showing high and low affinity transporter activity for cysteine and glycine in HBEC with the F508del^+/+^ mutation. The Hill1 equation was used to calculate the maximum effect (V_max_), substrate-transporter affinity (*K*_M_), and cooperativity of substrate binding (Hill coefficient, n) based on changes in benzamil-insensitive short-circuit current (*I*_sc_). (**A)** Saturation curves for cysteine suggesting uptake via a high-affinity transporter. (**B**) Saturation curves for cysteine exhibiting *I*_sc_ increases at higher concentrations, consistent with an additional low-affinity transport system. (**C**) Saturation curves for glycine suggesting uptake via a high-affinity transporter. (**D**) Saturation curves for lysine exhibiting *I*_sc_ increases at higher concentrations suggesting an additional low-affinity transport system. One to two independent experiments were conducted for each amino acid. Values are presented as mean ± SEM.

**Suppl. Figure 3:** Representative saturation kinetic plots showing high and low affinity transporter activity for cysteine and glycine in HBEC with the G542X^+/+^ mutation. The Hill1 equation was used to calculate the maximum effect (V_max_), substrate-transporter affinity (*K*_M_), and cooperativity of substrate binding (Hill coefficient, n) based on changes in benzamil-insensitive short-circuit current (*I*_sc_). (**A)** Saturation curves for cysteine suggesting uptake via a high-affinity transporter. (**B**) Saturation curves for cysteine exhibiting *I*_sc_ increases at higher concentrations, consistent with an additional low-affinity transport system. (**C**) Saturation curves for glycine suggesting uptake via a high-affinity transporter. (**D**) Saturation curves for lysine exhibiting *I*_sc_ increases at higher concentrations suggesting an additional low-affinity transport system. Two independent experiments were conducted for each amino acid. Values are presented as mean ± SEM.

**Suppl. Figure 4:** Representative current traces showing sequential inhibition of benzamil-insensitive short-circuit currents (*I*_sc_) using various anion channel blockers and a NKCC1 inhibitor **(A, C & E)** without prior blocking of SLC26A9 or stimulation of CFTR activity, and **(B, D & F)** without prior blocking of SLC26A9 but with prior stimulation of CFTR activity using forskolin in Wildtype-HBEC, and CF-HBEC with F508del^+/+^-, and G542X^+/+^mutations. Cells were treated with DMSO for 24 hours followed by exposure to Ringer’s solution or the selected amino acid formulation (SAA). After blocking ENaC with benzamil (6 µM; apical), the following inhibitors were added sequentially: 20 µM CFTRinh-172 (CFTR inhibitor; apical and basolateral), 20 µM CaCCinh-A01(ANO1 inhibitor; apical and basolateral), and 20 µM bumetanide (NKCC1 inhibitor; basolateral). In a second set of experiments, CFTR was stimulated with forskolin (cAMP activation; 10 µM, apical and basolateral) before blocking the various anion channels as described above.

**Suppl. Figure 5:** Changes in benzamil-insensitive short-circuit current (*I*_sc_) showing sequential inhibition of short-circuit *I*_sc_ by various anion channel blockers and an NKCC1 inhibitor without prior blocking of SLC26A9 in (**A**) wildtype-HBEC, and CF-HBEC with (**B**) F508del^+/+^-, (**C**) G542X/R785X, (**D**) W1282X/R1162X, and (**E**) G542X^+/+^ mutations in Ussing chambers.

Cells were treated with DMSO for 24 hours followed by exposure to Ringer’s solution or the selected amino acid formulation (SAA). After blocking ENaC with benzamil (6 µM; apical), and monitoring basal *I*_sc_ changes in lieu of blocking SLC26A9, the following inhibitors were added sequentially: 20 µM CFTRinh-172 (CFTR inhibitor; apical and basolateral), 20 µM CaCCinh-A01(ANO1 inhibitor; apical and basolateral), and 20 µM bumetanide (NKCC1 inhibitor; basolateral). Two to five independent experiments were conducted for each donor. Values are represented as mean ± SEM. Values for Ringer and SAA of each donor were compared using a two-sample t-test. A P-value of 0.05 was considered significant.

**Suppl. Figure 6:** Changes in benzamil-insensitive short-circuit current (*I*_sc_) showing cAMP-mediated stimulation of CFTR followed by sequential inhibition of short-circuit *I*_sc_ by various anion channel blockers and an NKCC1 inhibitor in (**A**) wildtype-HBEC, and CF-HBEC with (**B**) F508del^+/+^-, (**C**) G542X/R785X, (**D**) W1282X/R1162X, and (**E**) G542X^+/+^ mutations in Ussing chambers. Cells were treated with DMSO for 24 hours followed by exposure to Ringer’s solution or the selected amino acid formulation (SAA). After blocking ENaC with benzamil (6 µM; apical), CFTR was stimulated with forskolin (10 µM, apical and basolateral) followed by sequential addition of the following inhibitors: 20 µM CFTRinh-172 (CFTR inhibitor; apical and basolateral), 20 µM CaCCinh-A01 (ANO1 inhibitor; apical and basolateral), and 20 µM bumetanide (NKCC1 inhibitor; basolateral). Two to five independent experiments were conducted for each donor. Values are represented as mean ± SEM. Values for Ringer and SAA of each donor were compared using a two-sample t-test. A P-value of 0.05 was considered significant.

**Suppl. Figure 7:** Raw western blots to Fig. 9, A) F508del^+/+^ for CFTR protein, B) F508del^+/+^ for Calnexin used as loading control for A; C) G542X^+/+^ for CFTR protien, D) G542X^+/+^ Calnexin used as loading control for C; E) Western blot image for SLC26A9 in G542X^+/+^ and F508del^+/+^, F) Western blot image for β-Actin, used as loading control for E. G) Western blot image for ANO1 using HBEC with F508del^+/+^; H) Western blot image for β-Actin, used as loading control for G; I) Western blot image for ANO1 using HBEC with G542X^+/+^; and J) Western blot image for β-Actin, used as loading control for I.
