# Supplementary material for: Selective amino acid formulation enhances anion secretion and restores function in cystic fibrosis mutations
Source: Front Pharmacol. 2025 Aug 4;16:1522130. doi: 10.3389/fphar.2025.1522130 (PMC12358407; doi:10.3389/fphar.2025.1522130)
Supplement: Supplementary file 2 [file Presentation1.pptx]

## Slide 1
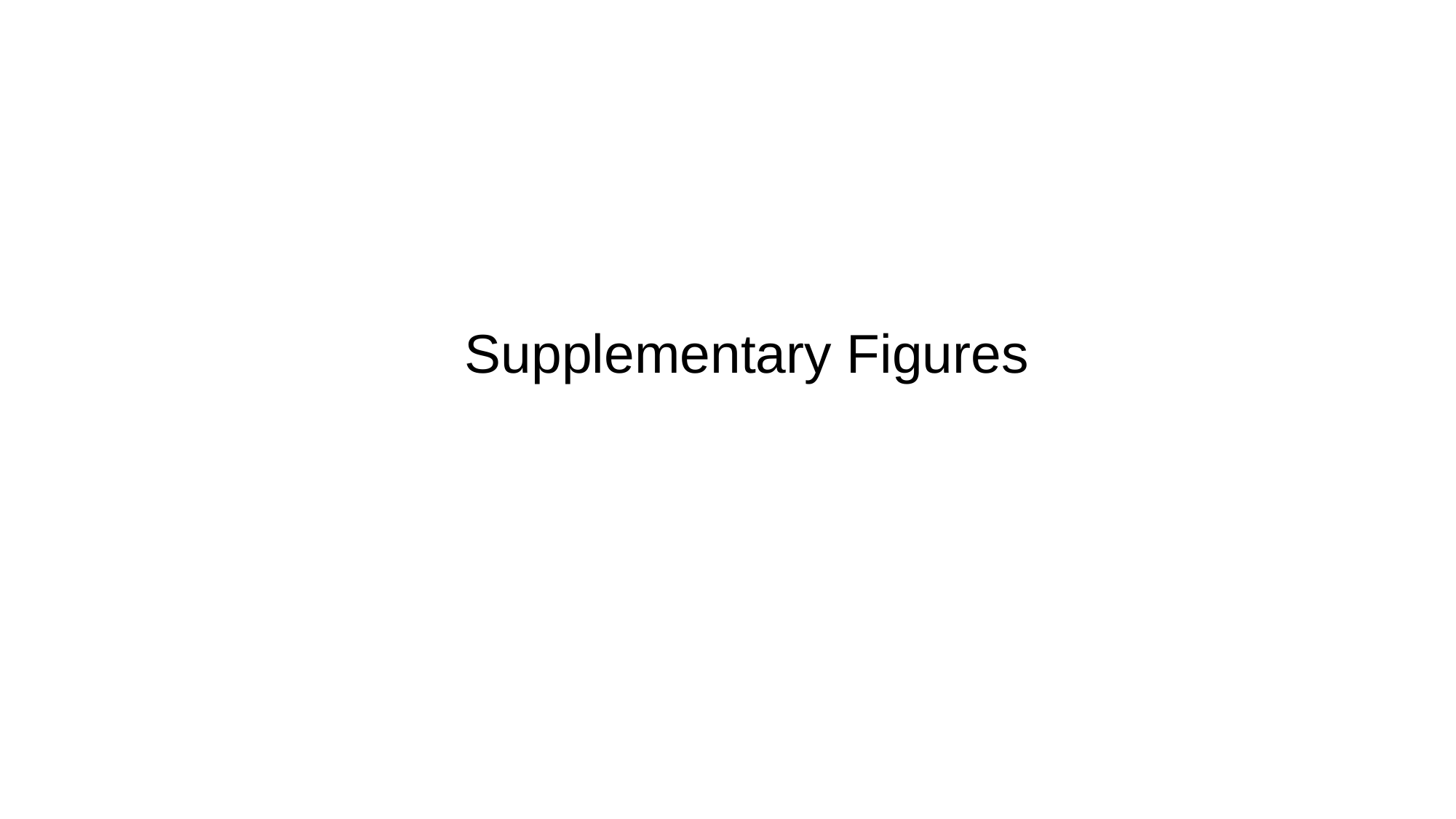

Supplementary Figures

## Slide 2
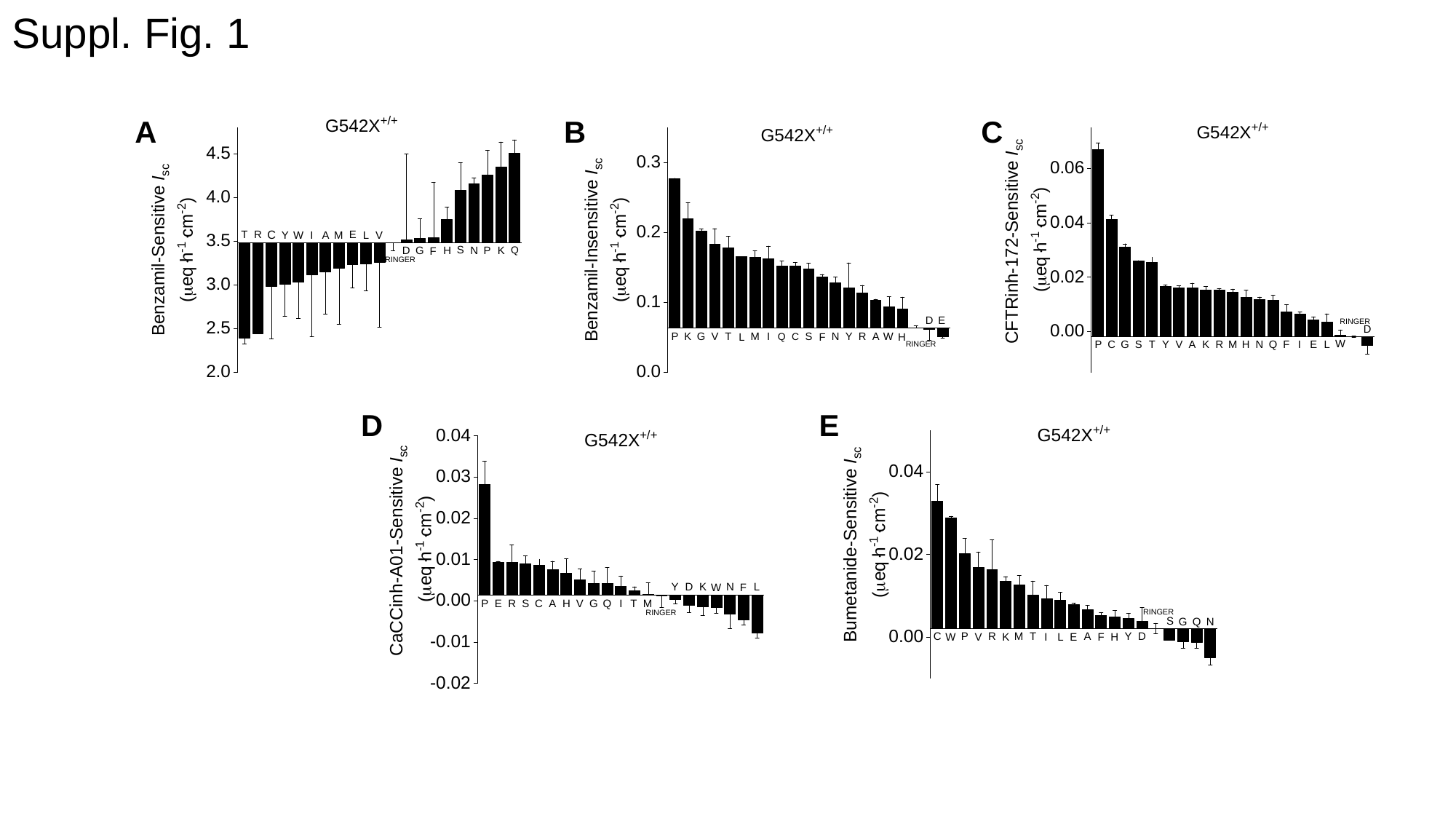

Suppl. Fig. 1
A
B
C
D
E

## Slide 3
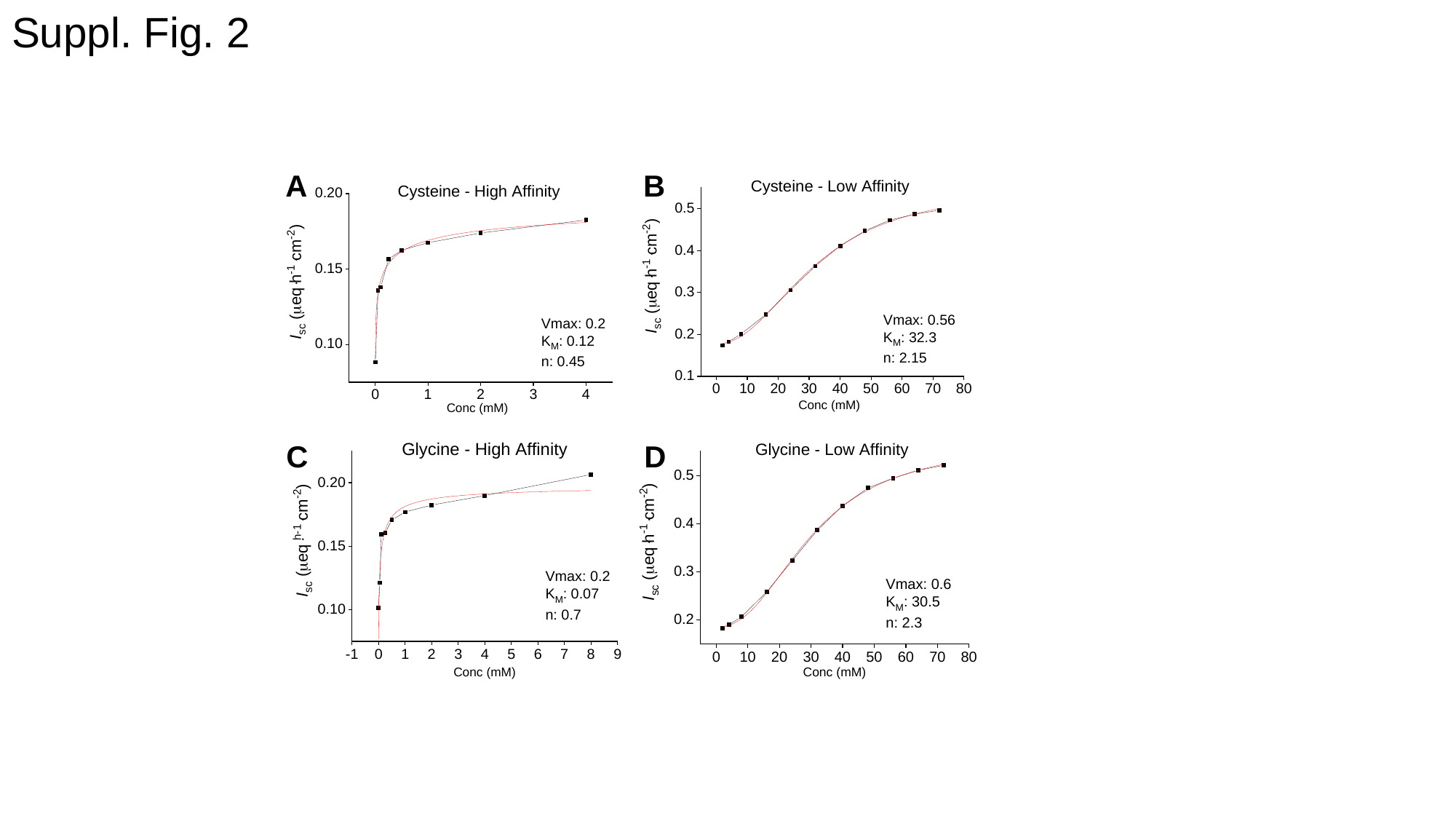

Suppl. Fig. 2
A
B
C
D

## Slide 4
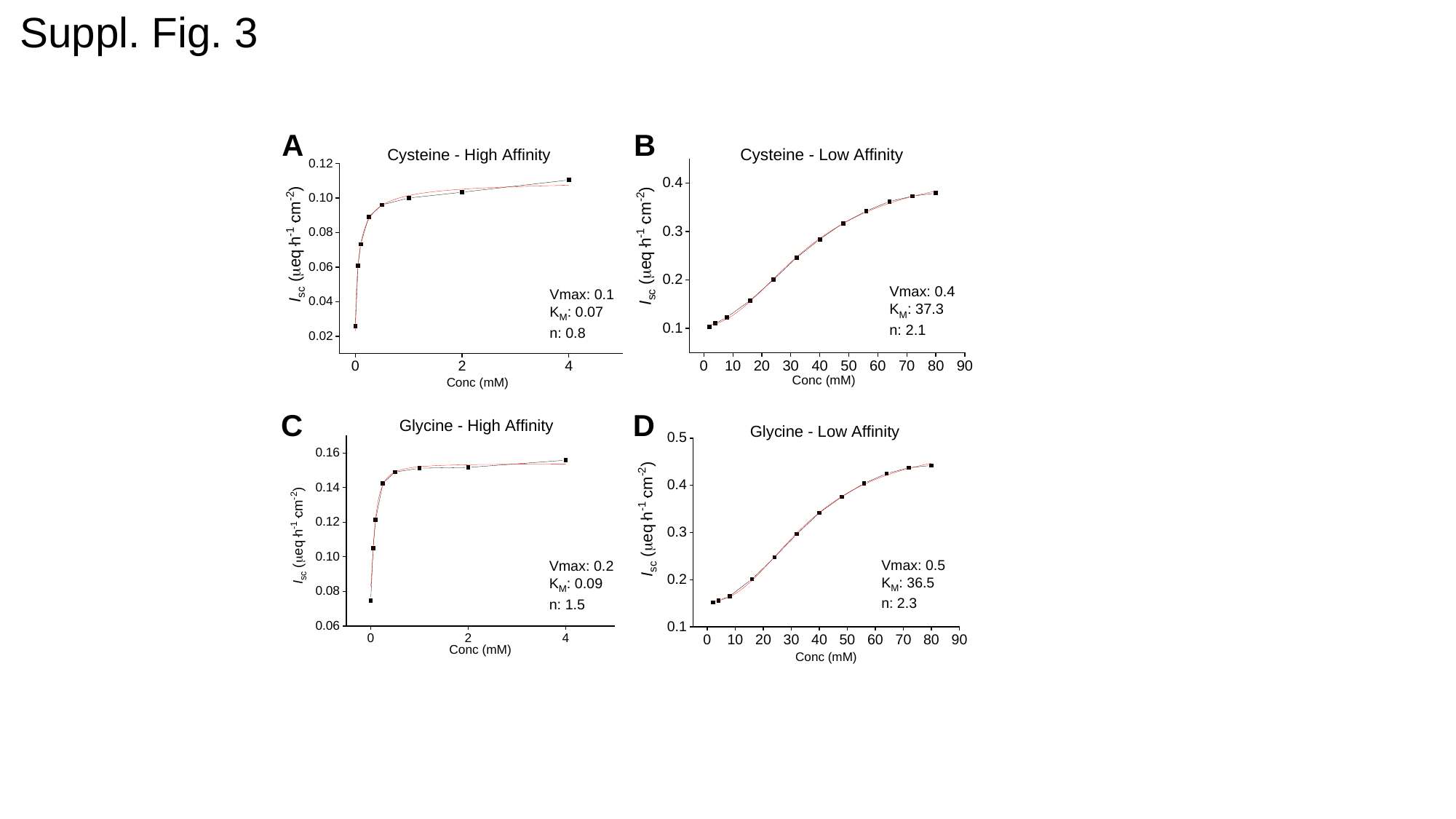

Suppl. Fig. 3
A
B
C
D

## Slide 5
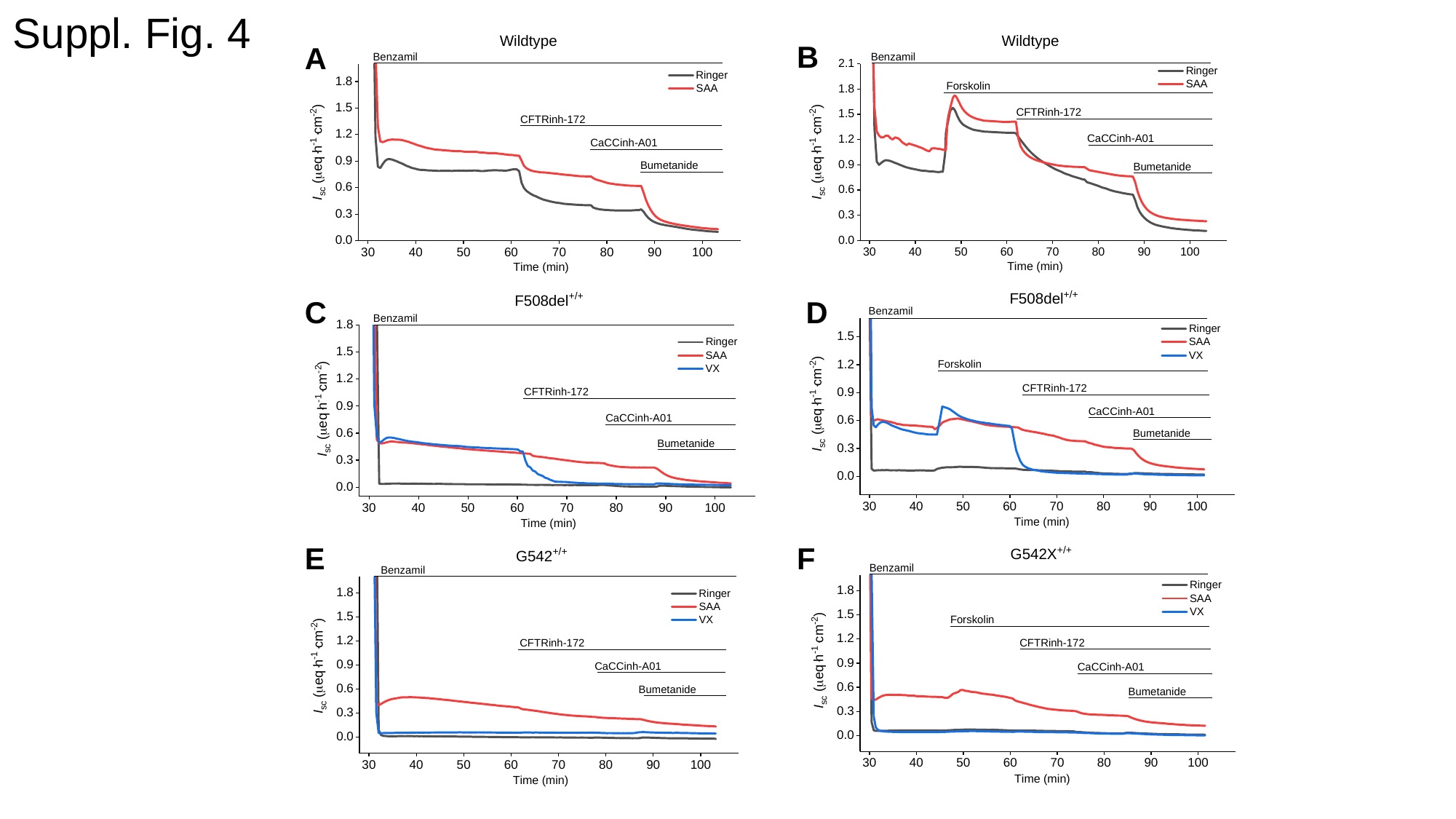

Suppl. Fig. 4
B
A
C
D
E
F

## Slide 6
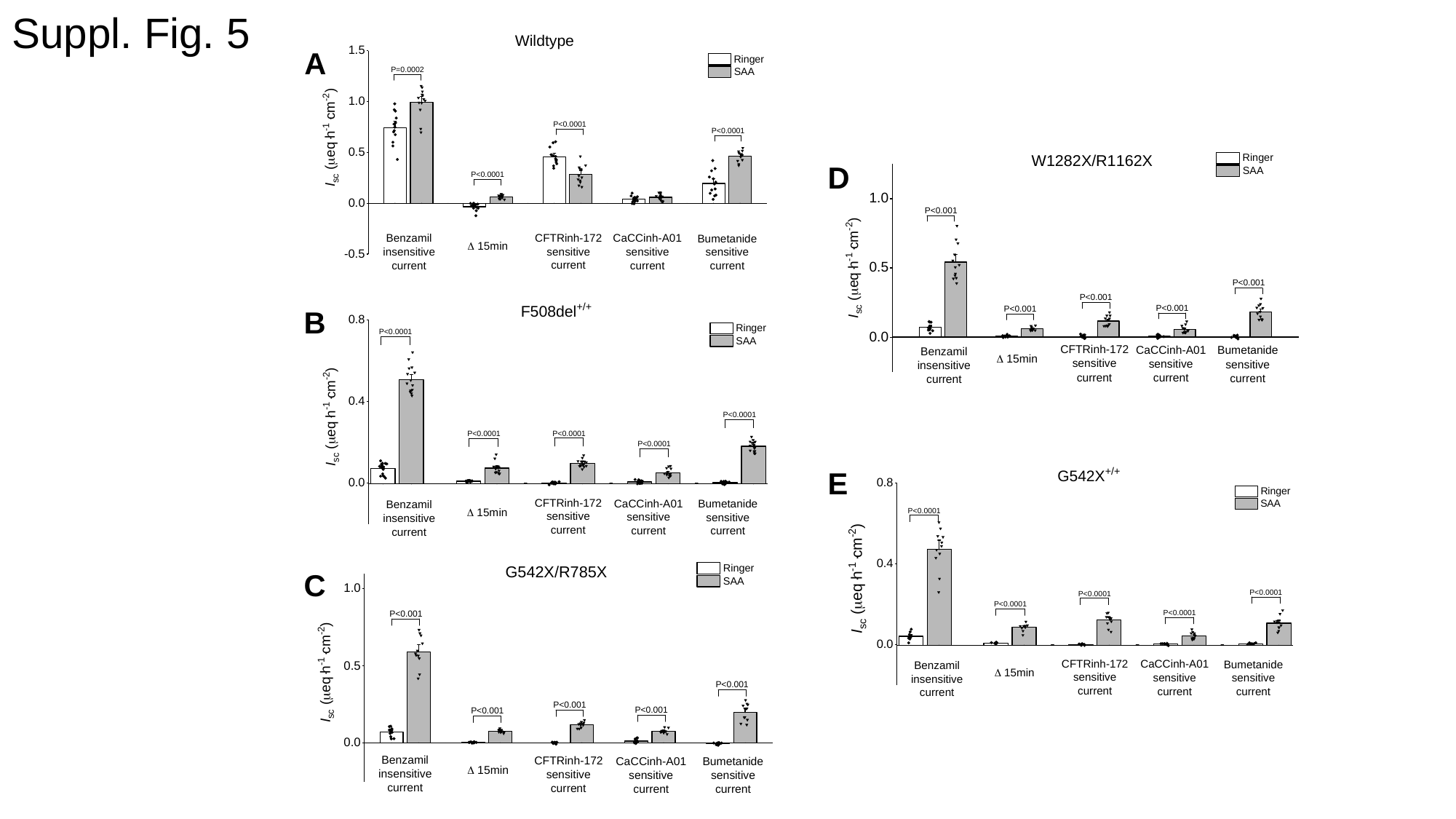

Suppl. Fig. 5
A
D
B
E
C

## Slide 7
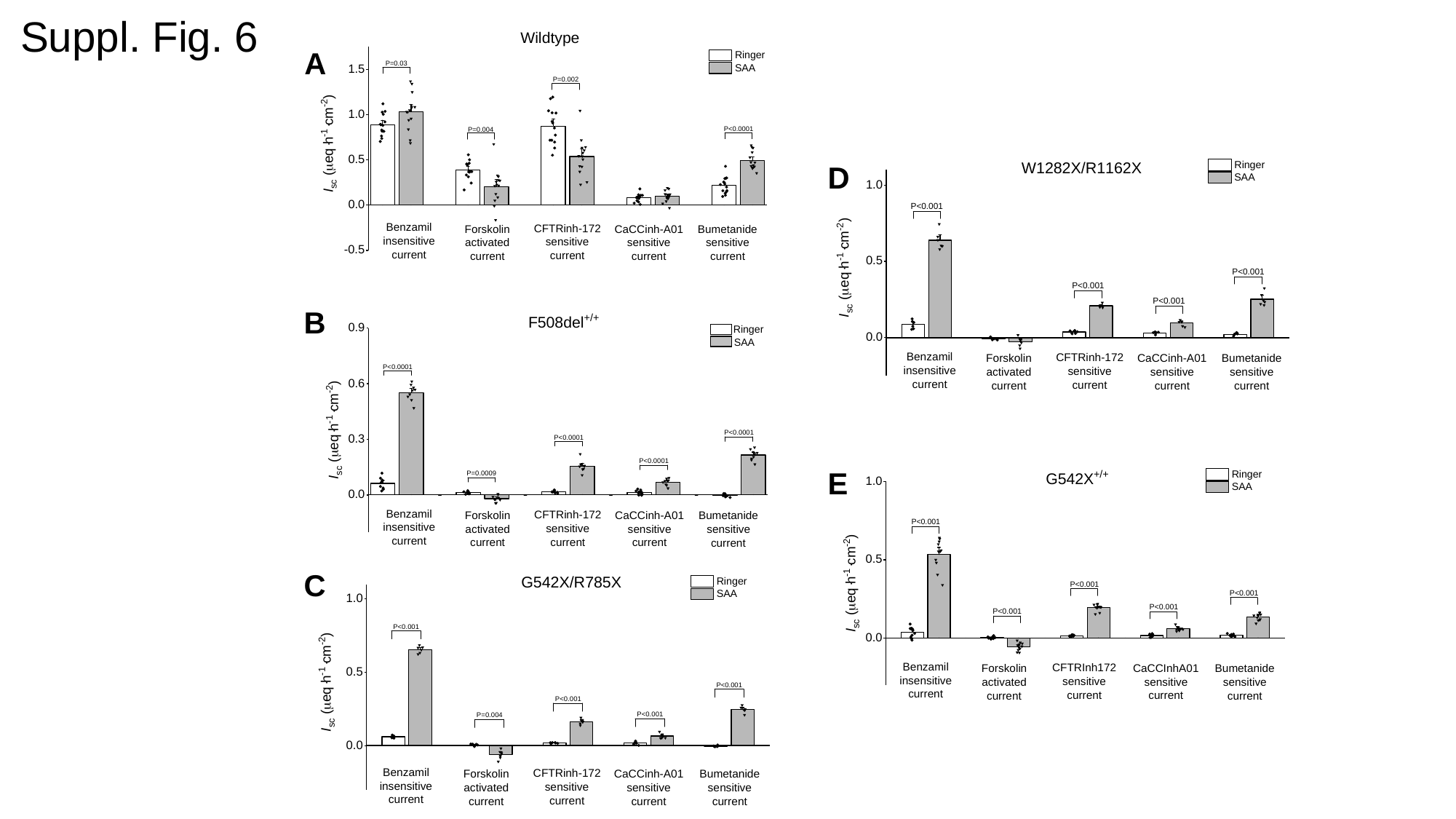

Suppl. Fig. 6
A
D
B
E
C

## Slide 8
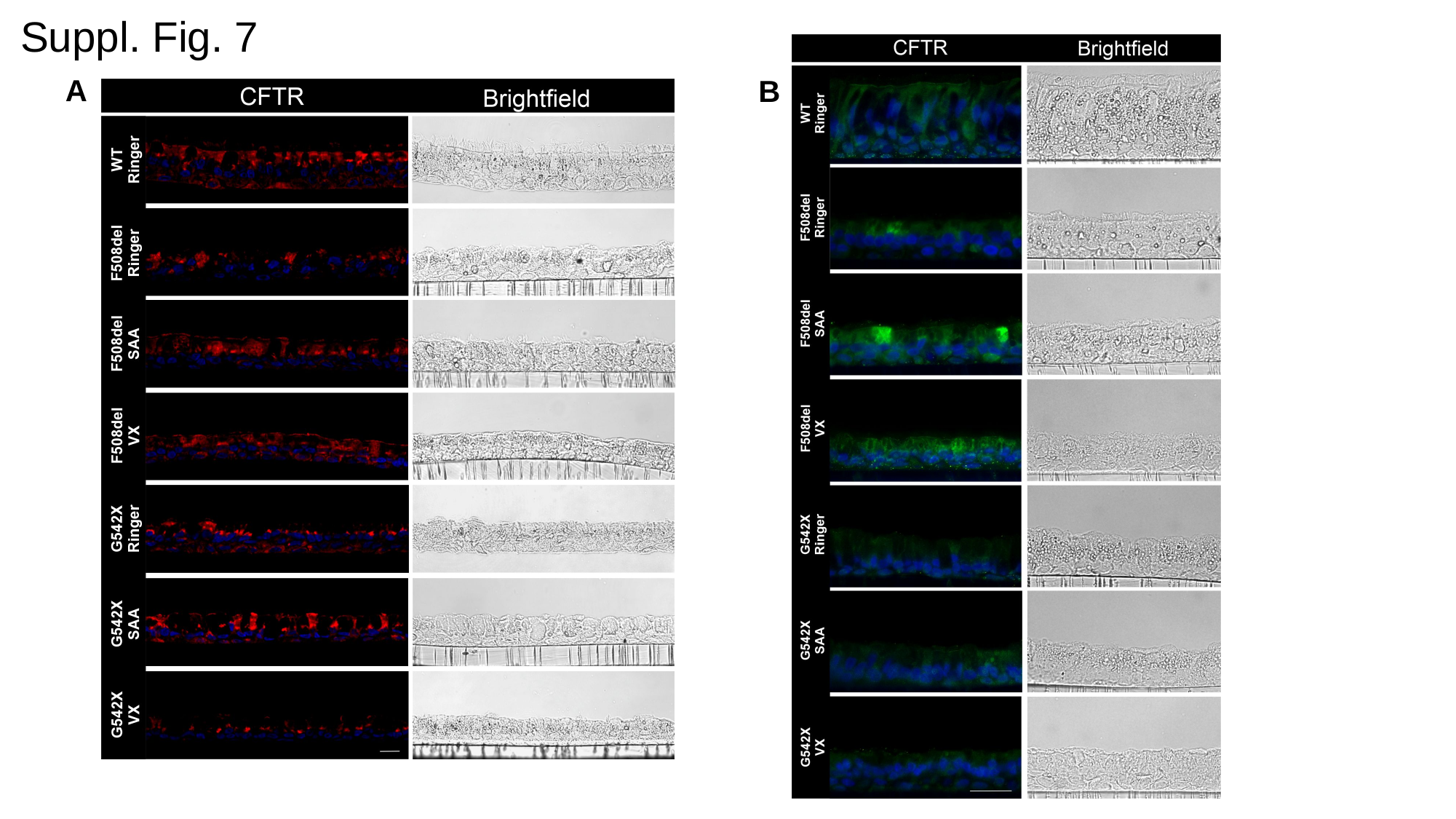

Suppl. Fig. 7
A
B
